# An Extended Binaural Real-Time Auralization System With an Interface to Research Hearing Aids for Experiments on Subjects With Hearing Loss

**DOI:** 10.1177/2331216518800871

**Published:** 2018-10-16

**Authors:** Florian Pausch, Lukas Aspöck, Michael Vorländer, Janina Fels

**Affiliations:** 1 Institute of Technical Acoustics, Teaching and Research Area of Medical Acoustics, RWTH Aachen University, Germany; 2 Institute of Technical Acoustics, RWTH Aachen University, Germany

**Keywords:** virtual acoustic environments, real-time auralization, binaural technology, room acoustics, hearing loss, hearing aids

## Abstract

Theory and implementation of acoustic virtual reality have matured and become a powerful tool for the simulation of entirely controllable virtual acoustic environments. Such virtual acoustic environments are relevant for various types of auditory experiments on subjects with normal hearing, facilitating flexible virtual scene generation and manipulation. When it comes to expanding the investigation group to subjects with hearing loss, choosing a reproduction system which offers a proper integration of hearing aids into the virtual acoustic scene is crucial. Current loudspeaker-based spatial audio reproduction systems rely on different techniques to synthesize a surrounding sound field, providing various possibilities for adaptation and extension to allow applications in the field of hearing aid-related research. Representing one option, the concept and implementation of an extended binaural real-time auralization system is presented here. This system is capable of generating complex virtual acoustic environments, including room acoustic simulations, which are reproduced as combined via loudspeakers and research hearing aids. An objective evaluation covers the investigation of different system components, a simulation benchmark analysis for assessing the processing performance, and end-to-end latency measurements.

## Introduction

The sense of hearing is an essential component for successful participation in social life. Approximately, 5% of the world’s population or 466 million people, 34 million children included, are affected by disabling hearing loss (HL) according to the World Health Organization (2017). Hearing aids (HAs) can help those affected to overcome challenging situations in their daily life. However, HA users often complain about the devices’ poor quality in such situations (Hougaard, 2011). One reason for this perceptual mismatch can be attributed to typical routines in the context of clinical diagnosis, where single or few loudspeakers (LSs) are used, only sometimes installed in an acoustically optimized hearing booth, playing back speech stimuli, and interfering broadband noise (ISO 8253-1, 2010; ISO 8253-2, 2009; ISO 8253-3, 2012; Katz, Medwetsky, Burkard, & Hood, 2014; Wagener, Brand, & Kollmeier, 1999). In addition, these routines are often based on simplistic acoustic stimuli such as pure tones and standardized words or phrases in quiet or noise (e.g., Kollmeier et al., 2011; Niklès & Tschopp, 1996). Such scenarios are therefore difficult to compare to real-life listening situations, where speech understanding in time-varying, noisy environments is required under conditions that can include adverse room acoustics. As a countermeasure to increase the satisfaction of HA users, real-life situations containing multiple static or moving sound sources under different room acoustic conditions have to be simulated to facilitate a more effective fitting process, So far, HAs fitted using existing clinical procedures are likely to require multiple visits to the audiologist for fine-tuning before achieving an “optimal” final setting.

Discrepancies between everyday-life listening and clinical fitting environments can undermine the overall effectiveness of HAs (Compton-Conley, Neuman, Killion, & Levitt, 2004; Cord, Baskent, Kalluri, & Moore, 2007; Walden, Surr, Cord, Edwards, & Olson, 2000). This situation has led to investigations of real-life performance of HA algorithms under challenging acoustic conditions, conducted by industrial companies and research groups in academia. In this context, the perceived performance or real-world benefit of, for example, directional HA microphone algorithms (Cord, Surr, Walden, & Olson, 2002; Gnewikow, Ricketts, Bratt, & Mutchler, 2009) has been evaluated applying virtual acoustic environments (VAEs). Since the simulated acoustic scene is probably closer to real-world conditions as a result of increased simulation and reproduction complexity compared with an oversimplified clinical fitting environment, this strategy potentially reduces the aforementioned gap. Assessing the performance of such HA algorithms can be carried out objectively through measurements taken from artificial heads (Grimm, Ewert, & Hohmann, 2015) or perceptually in experiments focusing, for example, on speech perception in noise (Cubick & Dau, 2016) or sound localization (Seeber, Baumann, & Fastl, 2004). In the last few years, such VAEs have been researched extensively and have qualified as a practical tool for creating complex acoustic scenarios in the study of auditory perception dealing with realistic listening situations in a laboratory-controlled environment (Cipriano, Astolfi, & Pelegrín-García, 2017; Grimm, Kollmeier, & Hohmann, 2016; Rychtáriková,Van den Bogaert, Vermeir, & Wouters, 2011; Seeber, Kerber, & Hafter, 2010; Zahorik, 2002). Increasing computational power and advanced simulation and convolution algorithms additionally allow for generating interactive scenarios with low latency (Mehra, Rungta, Golas, Lin, & Manocha, 2015; Noisternig, Katz, Siltanen, & Savioja, 2008; Pelzer, Aspöck, Schröder, & Vorländer, 2014; Schissler, Stirling, & Mehra, 2017; Wefers, 2015). Traditionally, reproduction of such VAEs relies on headphones (HPs), which restricts applications for HA users owing to feedback issues and uncontrolled behavior of HA algorithms. However, auralization systems reproducing VAEs are also capable of handling playback via LS set-ups through various technologies and have already demonstrated their suitability for the use in HA research (Grimm, Ewert, et al., 2015; Minnaar et al., 2013; Mueller, Kegel, Schimmel, Dillier, & Hofbauer, 2012; Oreinos & Buchholz, 2016). Regardless of the chosen LS-based reproduction technique, issues related to a proper integration of HAs into the VAE need to be resolved.

Representing one possible solution, an extended binaural auralization approach which has been developed to create VAEs especially for experiments on subjects with HL is presented here. To provide a rationale for selecting a reproduction system, this article starts with a synoptic overview of spatial audio reproduction technologies and their objective and subjective evaluation. Potential application areas of VAEs are discussed in the scope of auditory research and clinical practice. Thereafter, the concept and requirements for a system capable of generating and reproducing complex acoustic scenarios for people with HL are presented. The specific implementation of the concept focuses on configuration possibilities of the proposed system to create VAEs. Techniques for the simulation of room acoustics as well as HA signals and on how to properly combine the two simulation and playback paths in a hearing-aid auralization (HAA) module are presented. The application of a real-time auralization framework allows for real-world user movements which correspondingly update the simulation. In this context, signal processing strategies involving fast convolution algorithms, minimizing end-to-end latency (EEL), are discussed. Special focus is placed on the reproduction method of the acoustic signals and its evaluation. Simulated signals are reproduced combined via LSs and acoustic crosstalk cancellation (CTC) filters, emulating the reception of a surrounding sound field, and via the receivers of research hearing aids (RHAs), the latter signals being additionally processed on a real-time software platform for HA algorithm development before playback. In the following section on experimental methods, the measurements of spatial transfer functions, namely head-related transfer functions (HRTFs) and hearing aid-related transfer functions (HARTFs), are described, being the basis for both simulation paths in the HAA module and the key data for the benchmark analysis when evaluating an example scene. System components to be objectively investigated are introduced, such as properties related to the chosen HA fitting type, metrics quantifying the quality of LS-based reproduction, the example listening environment, and the method of measuring EEL. The results of these experimental evaluations are presented and subsequently discussed, ending with conclusions and outlook.

## Spatial Audio Reproduction Systems

### Previous Work and State of the Art

In VAEs, the main goal is to synthesize a specific sound environment, its acoustic properties included. Receivers and sound sources within such an environment are characterized by their directivities and movements on trajectories, leading to physical effects like Doppler shifts (Strauss, 1998; Vorländer, 2007). Reproduction of a synthesized virtual scene can be realized using different spatial audio reproduction approaches and set-ups, which vary in hardware requirements and complexity, and which have their benefits and drawbacks. Table 1 provides an overview of existing reproduction techniques. The listed systems can be roughly subdivided into two groups: Systems in the first category aim to create an authentic, that is, a physically correct, sound field (Blauert, 1997), whereas those in the second category aim to create a plausible, that is, a perceptually correct, sound field (Lindau & Weinzierl, 2012).

**Table 1.** Overview of Current Spatial Audio Reproduction Techniques Aimed to Create Physically Correct or Plausible Sound Fields.

Reproduction technique	Example systems and listening environments	Number of LSs	Benefits	Drawbacks
Binaural technology: Headphones, CTC (Blauert, 1997; Atal et al., 1966)	ITA,^a^ Virtual Acoustics (ITA Aachen, 2018; Wefers, 2015), RAVEN (Schröder, 2011); Sound Scape Renderer (Geier & Spors, 2012); TU Berlin,^j^ WONDER (WONDER Suite, 2017)	Small	Accurate sound source localization, physically correct sound field reproduction in a wide frequency range including room acoustic simulations, flexible source positioning, adaptive sweet spot through head tracking	Tracking system and measurement system for individual HRTFs or individualized HRTFs needed, high processing power for real-time room acoustic simulations needed, coloration artefacts in CTC playback, degraded channel separation especially in nonanechoic rooms
Discrete loudspeaker arrays	TUM,^b^ SOFE (Seeber et al., 2010)	Medium to high	Accurate sound source localization, listening with own HRTFs, possible simulation of room reflection patterns	Low simulation flexibility, modelling of “real” sound sources and simulated reflections at LS positions only
Panning techniques: VBAP, DBAP, MDAP (Pulkki, 2001), HOA (Daniel, 2000; Williams, 1999; Zotter, 2009); NFC-HOA (Daniel, 2003; Spors et al., 2011)	ITA,^a^ virtual reality laboratory (Pelzer, Sanches, Masiero, & Vorlände, 2011); SoundScape Renderer (Geier & Spors, 2012); IEM,^c^ CUBE (Zmölnig, Sontacchi, & Ritsch, 2003) and mAmbA (IEM mAmbA, 2014); CIRMMT,^d^ VIMIC (Peters, Matthews, Braasch, & McAdams, 2008); IRCAM,^e^ EVERTims (Noisternig et al., 2008), ESPRO 2.0 (Noisternig, Carpentier, & Warusfel, 2012); DTU,^f^ LoRA (Favrot & Buchholz, 2010); Hörtech,^g^ TASCARpro (Grimm, Luberadzka, Herzke, & Hohmann, 2015); HUT,^h^ DIVA (Savioja et al., 1999); T-Labs,^i^ Sound field synthesis toolbox (Wierstorf & Spors, 2012)	Medium to high	Flexible sound source positioning, possible simulation of reflection patterns based on VSSs, fair sound source localization and distance perception	VBAP or DBAP or MDAP: Plausible reproduction, possible discontinuities in VSS movements, increased apparent sound source width, distortion of binaural and monaural cues. (NFC-)HOA: upper frequency limit (spatial aliasing), pronounced sweet area, spectral imbalance, phase distortion, comb filter artefacts, simulation of near-by VSSs only in NFC-HOA systems.
Wave field synthesis (Ahrens, 2012; Melchior, 2011; Spors, 2005)	SoundScape Renderer (Geier & Spors, 2012); WONDER (WONDER Suite, 2017); T-Labs,^i^ Sound field synthesis toolbox (Wierstorf & Spors, 2012); Patent DE102007054152 A1 (Schulkrafft, 2002)	Medium to (very) high	Physically correct sound field reproduction up to a spatial aliasing frequency, large sweet area, flexible source positioning, fair localization and distance perception, multilistener suitability	Lack of height information for elevated VSSs, upper frequency limit (spatial aliasing), amplitude and truncation errors, coloration artefacts

*Note.* CTC = crosstalk cancellation; HRTF = head-related transfer function; LS = loudspeaker; VBAP = vector base amplitude panning; DBAP = distance-based amplitude panning; MDAP = multiple-direction amplitude panning; HOA = higher-order Ambisonics; NFC-HOA = near-field compensated higher-order Ambisonics; VSS = virtual sound source.

Example reproduction systems for each technique, including their benefits and drawbacks, are provided in addition to an estimation of the amount of LSs required. This table makes no claim to completeness.

a Institute of Technical Acoustics, RWTH Aachen University, Germany.

b Faculty of Electrical Engineering and Information Technology, Technical University of Munich, Germany.

c Institute of Electronic Music and Acoustics, University of Music and Performing Arts Graz, Austria.

d Centre for Interdisciplinary Research in Music Media and Technology, McGill University Montréal, Canada.

e Institut de Recherche et Coordination Acoustique/Musique, Paris, France.

f Department of Electrical Engineering, Technical University of Denmark, Lyngby, Denmark.

g HörTech gGmbH, Competence Center for Hearing Aid Technology, Oldenburg, Germany.

h Department of Computer Science, Helsinki University of Technology, Helsinki, Finland.

i Telekom Innovation Laboratories, Berlin, Germany. ^j^Technical University of Berlin, Germany.

Spatial audio reproduction systems based on binaural technology using HRTFs reproduce the source signal of a virtual sound source (VSS) in an acoustic environment physically correct at the ear drum of the listener (Blauert, 1997). Highest authenticity, with minimal influence of the reproduction device and the environment, can be most effectively achieved by playing back binaural signals over HPs using individual (Richter & Fels, 2016) or individualized HRTF data sets (Bomhardt & Fels, 2014) in combination with perceptually robust HP equalization (Masiero & Fels, 2011; Oberem, Masiero, & Fels, 2016; Pralong & Carlile, 1996). For binaural reproduction over LSs, a set of acoustic CTCs filters is usually applied (Atal, Hill, & Schroeder, 1966; Bauer, 1961). As no HPs are necessary, this reproduction approach potentially enhances the level of immersion and provides freedom of movement but also requires robust real-time signal processing and an optimized, ideally anechoic, listening environment.

Another way of creating authentic sound fields and simulating reflections relies on setups with a sufficiently high number of discrete LSs, where each sound source or reflection is represented by a single speaker (Seeber et al., 2010). This approach was used to measure localization performance in the horizontal plane of subjects either fitted with bimodal HAs or bilateral cochlear implants (Seeber et al., 2004).

Alternatively, three-dimensional (3D) LS arrays allow other approaches for reproducing plausible sound fields. As an extension of the classic stereophonic technique, phantom sources can also be generated three-dimensionally by driving a selected triplet of LSs. This technique is known as vector base amplitude panning (VBAP, Pulkki, 2001), distance-based amplitude panning (Pulkki, 2001), or, to ensure a more uniform panning, multiple-direction amplitude panning (Frank, 2014; Pulkki, 1999).

In a further panning approach, higher-order Ambisonics (HOA) is based on the decomposition of a surrounding sound field into a truncated series of frequency-independent spherical surface harmonics (Daniel, 2000; Williams, 1999; Zotter, 2009). The number of LSs and the decoder strategy (Zotter & Frank, 2012; Zotter, Pomberger, & Noisternig, 2012) define the perceptual quality and the upper frequency limit of a synthesized sound field, which is restricted to a specific area or sweet spot. Only setups with near-field compensated HOA allow for the reproduction of close-by sound sources (Daniel, Moreau, & Nicol, 2003; Spors, Kuscher, & Ahrens, 2011). Different realizations of HOA systems have already been applied to HA-related research (e.g., Favrot & Buchholz, 2010).

Wave field synthesis (WFS) is a reproduction technique requiring a similar amount of hardware compared to HOA when aiming at the same sound field accuracy in a given listening area. Sound field synthesis is achieved through superposition of elementary spherical waves (Melchior, 2011; Spors, 2005). The advantage of this technique is the reproduction of physically correct sound fields up to a certain spatial aliasing frequency in an extended sweet spot area (Daniel et al., 2003; Spors & Ahrens, 2007), making it suitable for multiple listeners. A WFS system was used by Schulkrafft (2002) for the fitting of HAs, measuring the subject’s audiogram with HAs attached.

### Evaluation and Reproduction Errors of Spatial Audio Reproduction Systems

To obtain well-grounded and accurate experimental results in auditory research, available spatial audio reproduction systems have to be evaluated on different objective and perceptual levels. Possible measures for quantifying the sound field error between synthesized and reference sound fields include, among others, the analysis of room acoustic parameters such as reverberation times *EDT* and *T*_30_ or clarity indices like *C*_50_ and *C*_80_, long-term power spectral density of the HA microphone signals, binaural parameters such as (mean) interaural time and level differences, interaural cross correlation coefficient, and the improvement in signal-to-noise ratio using multichannel HA algorithms (cf. Cubick & Dau, 2016; Grimm, Ewert, et al., 2015; Oreinos & Buchholz, 2014, 2016). For perceptual system evaluations, a road map to assess spatial sound perception was proposed by Nicol et al. (2014), including methods aiming at measuring perceptual errors, for example, by rating the difference between reference and reproduced sound samples. Similar spatial audio quality parameters were discussed and provided by Lindau et al. (2014). In addition to thorough objective evaluations of reproduction systems, this well-defined vocabulary can be applied for subjectively rating reproduction quality.

Several research groups investigated authenticity and plausibility of binaural technology using HPs (e.g., Lindau, Hohn, & Weinzierl, 2007; Lorho, 2010; Oberem et al., 2016; Pike, Melchior, & Tew, 2014). In binaural reproduction via LSs, the reduced channel separation (CS) caused, amongst other reasons, by unwanted room reflections in typical listening environments limits the binaural signal reproduction fidelity (Kohnen, Stienen, Aspöck, & Vorländer, 2016; Sæbø, 2001). In addition, Majdak, Masiero, and Fels (2013) showed a significant effect of nonindividualized HRTF data sets on localization performance. Also, latency introduced by tracking systems when updating auralization based on the listener’s real-world position is highly relevant to any dynamic binaural reproduction system (Brungart, Simpson, & Kordik, 2005; Lindau, 2009; Yairi, Iwaya, & Suzuki, 2006).

Panning approaches such as VBAP and its variants are mostly used for artistic applications, in theaters, or in entertainment (Pulkki & Karjalainen, 2008). Limitations in VBAP can be traced back to increased apparent source width as well as the distortion of binaural and monaural cues (Pulkki, 2001) which potentially affect localization of VSSs in the sagittal plane (Baumgartner & Majdak, 2015). However, owing to possibilities of modeling reflections as VSSs, VBAP represents one approach for the creation of VAEs (Savioja, Huopaniemi, Lokki, & Väänänen, 1999), in combination with room acoustic simulations (Pelzer, Masiero, & Vorländer, 2014). Due to the coloration potential of VBAP (Frank, 2013), nearest neighbour panning can be considered as alternative option for reproducing reflections.

Examples of reproduction errors in systems based on HOA include distorted information above a certain spatial aliasing frequency (Spors & Ahrens, 2007), spectral imbalance (Daniel, 2000), potentially perceivable phase distortions, or comb-filter artifacts (Frank, Zotter, & Sontacchi, 2008; Solvang, 2008), which can possibly be diminished because of the influence of the listening environment (Santala, Vertanen, Pekonen, Oksanen, & Pulkki, 2009). Specific evaluations of this reproduction technique were conducted, for example, by Oreinos and Buchholz (2015) and Grimm et al. (2016).

Shortcomings of WFS systems include amplitude errors caused by secondary sound source mismatch (Ahrens & Spors, 2009), truncation errors owing to finite LS array dimensions (Berkhout, de Vries, & Vogel, 1993), and spatial sampling of theoretically continuous LS arrays (Spors & Ahrens, 2009). The latter leads to additional wave front components above the spatial aliasing frequency, thus distorting spatial and spectral fidelity of the target sound field and producing coloration artifacts (Wierstorf, 2014).

In summary, different reproduction approaches and systems have been researched intensively in the recent past. To obtain practical results from such evaluations, it is important to support computer simulations by means of measurement-based strategies with the aim of quantifying in-situ system limitations with regard to reproduction fidelity and sound field errors. Such combined evaluation strategies should preferably be merged in a road map providing common ground for standardized quality assessments of spatial audio reproduction systems.

### Potential Application Areas of VAEs Reproduced by Spatial Audio Reproduction Systems

Regardless of the chosen spatial audio reproduction technique, flexibility is crucial when creating VAEs, providing for applications in auditory research, clinical practice, and auditory training. In the following, some potential application areas are outlined.

To measure speech perception in different spatial configurations of a target and a distractor talker, well-established paradigms such as the listening in spatialized noise-sentences test by Cameron and Dillon (2008) can be conducted under plausible room acoustic simulations (Pausch, Peng, Aspöck, & Fels, 2016). Human behavior can be investigated with potentially increased validity, given the opportunity of simulating static or dynamic VSSs (Lundbeck, Grimm, Hohmann, Laugesen, & Neher, 2017) moving on predefined trajectories and including source directivities, simulated in virtual rooms. Such investigations may include behavioral experiments on selective auditory attention, being of multidisciplinary interest (Lawo, Fels, Oberem, & Koch, 2014; Oberem, Lawo, Koch, & Fels, 2014). In this context, several studies confirm detrimental effects of noise on cognitive tasks (for a review see, e.g., Hellbrück & Liebl, 2008; Szalma & Hancock, 2011), especially in children (Klatte, Bergström, & Lachmann, 2013; Vasilev, Kirkby, & Angele, 2018). Controlled noise scenarios with calibrated playback levels can be realized using VAEs because of their flexibility when creating VSSs with arbitrary source signals and the possibility of reproducing physical effects such as Doppler shifts and room reflections (Vorländer, 2007).

As initial intervention to compensate for the consequences of HL (Tambs, 2004), the patient is, for example, fitted with an HA following standard gain prescription rules based on individual audiograms (Keidser, Dillon, Flax, Ching, & Brewer, 2011; Kiessling, 2001). After several visits to the audiologists, presumed optimal settings are found (Kochkin et al., 2010), however, especially older adults often complain about the mismatch between expectation of the clinical fitting outcomes and using their HAs in real-world, noisy listening situations (Hougaard, 2011). Overall poor satisfaction with their fitted HAs may lead to untreated HL (i.e., nonuse of the HAs), putting older adults at much higher risks of dementia and accelerated cognitive decline (Li et al., 2014; Lin et al., 2011, 2013). The integration of VAEs allows for more flexible and efficient procedures in audiological diagnosis by supplying an interface to HAs. Subsequent HA fitting can then be conducted in VAEs and will likely decrease discrepancies between laboratory and real-life performance (Compton-Conley et al., 2004).

Alongside the prescription of HAs, another important step facilitating daily communication in the hard of hearing population and stimulating accelerated integration into social life involves individually designed rehabilitation programs providing auditory training (Bettison, 1996; Henshaw & Ferguson, 2013; Sweetow & Palmer, 2005). In this context, VAEs can be used as testing and training environments to treat, for example, the negative effects of spatial processing disorder as part of central auditory processing disorder (Cameron, Glyde, & Dillon, 2012; Musiek & Chermak, 2013).

## Concept of an Extended Binaural Real-Time Auralization System

This section presents an auralization system which was designed and developed for the aforementioned application areas. Technical requirements are defined according to selected research paradigms and desired scenarios for auditory diagnosis and training procedures involving subjects with HL. To fulfill these requirements, a general concept of an extended binaural real-time auralization system has been developed. The system offers full control over simulation and reproduction of realistic VAEs, including room acoustic simulations, while providing a high degree of flexibility for the definition and manipulation of virtual acoustic scenes.

**Figure 1.**
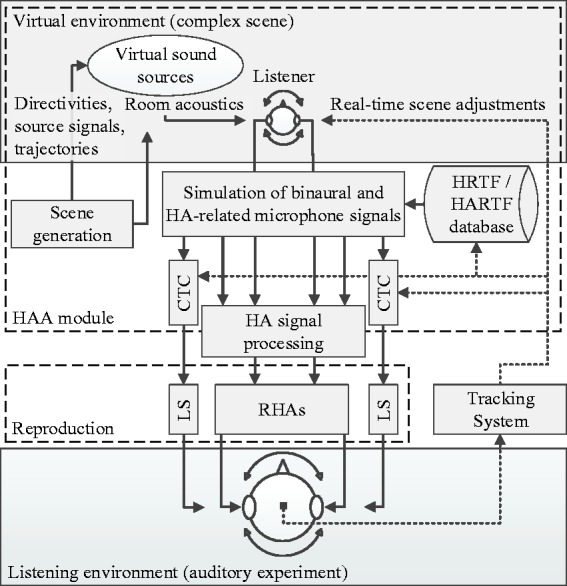
The concept of an extended binaural auralization system. The upper block shows elements crucial for generating a complex scene by means of a virtual environment. Such a complex scene, set in a virtual room with simulated room acoustics, includes static or moving virtual sound sources with inherent source directivities, playing back arbitrary source signals. The simulation of binaural signals is based on databases of HRTFs and HARTFs. For external sound field simulation, binaural signals are processed on the basis of acoustic CTC filters and played back to the listener via LSs, installed in the listening environment used for auditory experiments. The simulated HA signals are processed by HA algorithms and reproduced via RHAs, worn by the listener. To enable virtual scene updates according to the listener’s head position and orientation, a motion tracking system is integrated. *Note.* HAA = hearing aid auralization; HA = hearing aid; CTC = crosstalk cancellation; HRTF = head-related transfer function; HARTF = hearing aid-related transfer function; LS = loudspeaker; RHA = research hearing aid.

The idea of the proposed system is shown in Figure 1. Based on the concept of binaural rendering, the subject listens to a complex virtual scene. This scene, either replicating outdoor or indoor scenarios, is denoted as *complex* since it contains multiple static or moving VSSs. Each VSS is characterized by its source power level, its directivity, and its location or movement on predefined trajectories. The environment in which the acoustic scene takes place is represented by a 3D model and can easily be created using conventional computer-aided design software. Room acoustic simulations, based on given geometry, absorption, and scattering characteristics of surface materials used in the model, increase the degree of plausibility. These surface materials for the 3D model are selected according to desired room acoustic conditions, for example, by defining a target reverberation time. A typical indoor scene, set in a restaurant, will be presented in the Experimental Methods section to be applied within the scope of the benchmark analysis of the acoustic simulation.

Room acoustic simulations use a database of spatial transfer functions. All sound propagation paths from each VSS to the receiver are represented by impulse responses, also containing receiver characteristics. These impulse responses are generated separately as binaural filters with respect to the listener’s ear canal entrance, based on HRTFs, or as filters with respect to HA sensors, based on HARTFs. HRTFs can either be acquired through measurements from individuals with open or blocked meatus (Oberem et al., 2016) using a fast measurement system (Richter & Fels, 2016), from an artificial head (Algazi, Duda, Thompson, & Avendano, 2001; Gardner & Martin, 1995; Schmitz, 1995), by individualizing generic data sets through incorporating anthropometric data (Bomhardt & Fels, 2014), or by means of numerical simulations (Fels, Buthmann, & Vorländer, 2004; Katz, 2001). Similarly, HARTF data are acquired through measurements of direction-dependent transfer functions at the HAs’ microphone positions (Kayser et al., 2009; Denk, Ernst, Ewert, & Kollmeier, 2018). Convolving the impulse responses, merged with room acoustic filters, with anechoically recorded sound files (Vorländer, 2007) results in binaural head-related and HA-related signals.

To allow for user interaction, the movement of the listener in real world is captured through motion tracking. This makes it possible to adjust the virtual scene in real time and ensures that VSSs stay in position in case of head rotation or displacement (Savioja et al., 1999). As binaural signal reproduction is intended for subjects with mild to moderate HL with residual hearing, and a control group with normal hearing (NH), the sound field arriving at the subjects’ eardrums must also be accurately simulated and reproduced. Binaural LS-based reproduction favors natural sound field perception as opposed to binaural listening over HPs. To this end, an acoustic CTC filter network for transaural reproduction is applied (Masiero, 2012) and continuously updated, following the listener’s position and orientation as captured by the motion tracking system.

For avoiding biased results due to different proprietary HA algorithms and HA models, the listener is equipped with a pair of RHAs. This allows direct input of simulated audio signals, which are then reproduced by the RHAs’ receivers. Before HA-related signals are sent to the HAs, they are processed by a real-time software module, that is, a master hearing aid (MHA) software platform (e.g., Curran & Galster, 2013; Grimm, Herzke, Berg, & Hohmann, 2006) emulating typical HA algorithms. The number of MHA input channels can be configured according to the paired microphone count of the virtual HAs, ranging from two to six channels in current HA models. In addition to binaural LS-based reproduction, the two output signals of the MHA are finally played back by the receivers of the RHAs. As the RHAs’ microphones are not used during reproduction, no adaptive feedback cancellation is necessary. If, however, feedback simulation is required, the MHA’s output could be re-routed to its input, while also considering the impulse response of the feedback path.

## System Requirements

### Simulation of Complex Acoustic Scenes

Simulation models have to provide plausible cues for the spatial distribution of multiple VSSs, their source characteristics, such as level and directional properties, as well as reflections, determined by geometrical and acoustic properties of the virtual scene. For plausible sensation, tracking of user movement is crucial and requires at least part of the simulation being executed during the run-time of the program, necessitating efficient signal processing and state-of-the-art processing power, especially if the scene contains multiple VSSs. A good trade-off between simulation accuracy and processing workload can be achieved by utilizing geometrical acoustics simulation models (Kuttruff, 2016), allowing for accurate results in real time (Aspöck, Pelzer, Wefers, & Vorländer, 2014). Although sufficient computational power must be provided for reproducible real-time signal processing in different experimental sessions, the system should be feasible for use on desktop computers to be affordable for research institutions.

### Processing of HA Signals

Subjects with HL should be supplied with HA signals in a transparent and controlled way, without introducing any bias caused by differences in proprietary HA algorithms. Therefore, similar to conventional HRTF measurements (Møller, Sørensen, Hammershøi, & Jensen, 1995), spatial transfer functions of behind-the-ear (BTE) HA microphones have to be measured. These HARTFs must be integrated for each VSS during the propagation simulation. Simulated HA input signals should be processed by an HA algorithm software toolbox, allowing the researcher to control and modify the HA fitting. Depending on the focus of research, these algorithms do not have to provide the full function range of modern HAs, but should, at least, feature established fitting protocols and spatial processing techniques such as dynamic range compression or beamforming. Fitting protocols and the preparation of the HA algorithm software toolbox should be carried out in cooperation with audiologists, the selection of audiological data sets to be used in simulation environments should, however, be a manageable task for researchers with only limited background in audiology.

### Combined Binaural Reproduction

#### HA-based reproduction

To insert and reproduce simulated HA signals, the RHA must feature direct audio input and a receiver unit. The ear piece ideally allows subjects to use their residual hearing capabilities to a high extent. Controlling the delay of HA-based reproduction relative to external sound field playback needs to be provided, either before or after HA signal processing.

#### External sound field reproduction

Reduction of acoustic crosstalk in the LS-based binaural reproduction, used for external sound field simulation, can be achieved by using a CTC system consisting of two or more LSs (Atal et al., 1966; Bauck & Cooper, 1992). The number and positioning of LSs determine the system’s stability in case of user rotation. If only two LSs at azimuth angles^1^ of, for example, ϕ=±45deg, are used, the system will suffer from instabilities if the user’s viewing direction is within a critical angular range of LS positions and outside the LS span angle (Lentz, 2008). Therefore, a recommended minimum number of three to four LSs should be included. With regard to the distance between LSs and listener, LS dimensions should be small in relation to listening distance (Guang, Fu, Xie, & Zhao, 2016). In addition, LSs should be installed in elevated positions with regard to the horizontal plane, intersecting the listener’s head center at ear axis height, which will result in less pronounced notches and azimuthal variations in playback HRTF data (Parodi & Rubak, 2010).

#### Listening environment

Regarding the listening environment, the spatial audio reproduction system should be set up in an optimized but comfortable laboratory. On the one hand, the room must provide adequate acoustic conditions with low background noise level (BNL), complying, for example, with ANSI/ASA S3.1 (1999), and minimal impact of room acoustics to avoid excessive room reflections which would considerably degrade the fidelity of external sound field reproduction (Ward, 2001). On the other hand, the room’s environmental conditions like temperature or fresh air supply should be stable throughout experiments, preferably with low visual distraction potential and attachment facilities for technical equipment, such as LSs and electromagnetic or optical motion tracking systems. Although electromagnetic motion tracking systems can be installed invisibly, they rely on signal transmission via cables. To avoid restricted mobility on account of its wiring, optical variants of the motion tracking system should be preferred.

### System Latency

EEL is a crucial parameter in every real-time auralization system and directly determines its reactivity and effectiveness in generating presence (Slater, Lotto, Arnold, & Sanchez-Vives, 2009). In this article, dynamic EEL is defined as time difference between the time instance when the real-world user position is changing, for example, as a result of head rotations or translations, and the time instance when the updated auralized sound arrives at the listener’s ear drums. Achieved dynamic EEL should be on average below detectable thresholds of 60 to 75 ms, as reported by Brungart et al. (2005) and Yairi et al. (2006), using different source signal types and measurement methods. Lindau (2009) reported higher pooled threshold values (*M* = 107.63 ms, *SD* = 30.39 ms) with no observable effect, neither for auralization of anechoic or reverberant VAEs, nor for different stimuli, that is, noise, music, and speech.

## Specific Implementation

**Figure 2.**
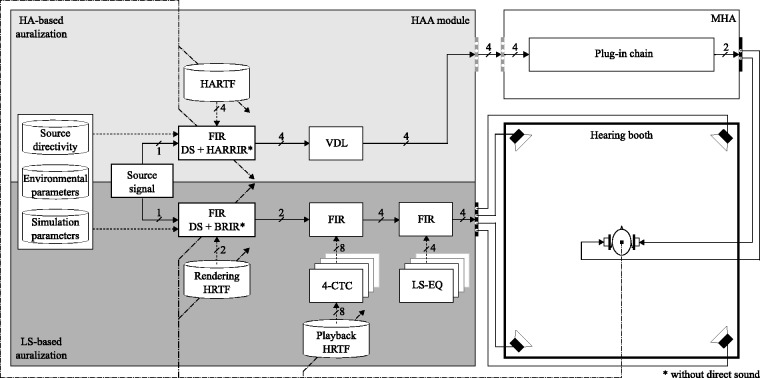
Schematic depiction of the implemented extended binaural real-time auralization system, consisting of HA-based (light gray area) and LS-based (dark gray area) binaural auralization paths with acoustic CTC filter network (4-CTC) and LS-EQs. Both auralization paths are contained in the HAA module. The DS of binaural signals is generated by applying filters based on HARTF and HRTF. Results of room impulse response simulations, neglecting DS, are represented by HARRIRs and BRIRs, which both rely on databases for source directivity, environmental and simulation parameters. The HA-based binaural auralization is time-delayed using a VDL to account for typical real-life HA latencies. Before being played back over RHAs, delayed HA signals are further processed on a MHA software platform, emulating HA algorithms comprised by a plug-in chain. Subjects can also utilize residual hearing capabilities by listening to a simulated external sound field played back through a set of LSs, preferably installed in a hearing booth. For dynamic reproduction, the subject’s real-world head position and orientation are captured by an optical motion tracking system, which initiates real-time updates of relevant filter sets (dash-dotted parameter signal). All filtering operations are realized via FIR filters. Arrows with dashed lines indicate parameter signals to update the selection of transfer functions and coefficients of associated FIR filters. Gray outlet or inlet boxes denote software connections, whereas black outlet boxes denote physical hardware outputs. *Note.* HA = hearing aid; HARTF = hearing aid-related transfer function; FIR = finite impulse response; DS = direct sound; HARRIR = hearing aid-related room impulse response; VDL = variable delay line; BRIR = binaural room impulse response; HRTF = head-related transfer function; CTC = crosstalk cancellation; LS = loud speaker; LS-EQ = loudspeaker-equalization filter; HAA = hearing aid auralization; MHA = master hearing aid.

Based on its concept and with the aim of fulfilling the presented set of requirements, an extended binaural real-time auralization setup was implemented, as schematically shown in Figure 2, including major signal processing stages. On the left, the HAA module consisting of HA-based and LS-based auralization paths is depicted. For generating the binaural signal’s direct sound (DS) in the respective auralization path, spatial transfer function databases, namely either rendering HRTF or HARTF data sets, measured from an artificial head or individually and stored efficiently in OpenDAFF (2018) format, are accessed. Results of room impulse response simulations, neglecting the DS, are defined as hearing aid-related room impulse responses (HARRIRs) and binaural room impulse responses (BRIRs) and combined with the respective HRTF or HARTF data set in a finite impulse response filter. Databases covering source directivities, environmental parameters such as temperature or humidity, simulation parameters, as well as source trajectories for the simulation of moving VSSs are additionally integrated. The HA-based binaural signal is time delayed, relative to the LS-based binaural signal, using a variable delay line which accounts for typical real-life HA latencies (Stone, Moore, Meisenbacher, & Derleth, 2008). Binaural signals for the external sound field simulation in the LS-based path are processed by acoustic CTC filters, implemented as 4-CTC (Masiero, 2012). CTC filters are calculated from a database of generic or individual playback HRTFs. Before playing back binaural signals, the spectral influence of the LS transducers are minimized by applying LS equalization filters, representing inverse, on-axis, free-field LS transfer functions. Subset selection of spatial transfer functions from the respective database and room acoustic filters is additionally determined by the listener’s current position and orientation, which both are continuously captured by a motion tracking system and fed back to the HAA module, as shown by dash-dotted feedback lines.

On the top right, the signal processing plug-in chain of the MHA is shown, consisting, for example, of filter banks, dynamic range signal processing algorithms or beamforming implementations. As input signals to the MHA, HA-related output signals of the HAA module are sent back via low-latency software loop-back. In the current system realization, the MHA must be capable of processing a maximum number of four input channels, depending on the microphone channels available on the RHAs which had been previously used for spatial transfer function measurements.

Both binaural signals are finally played back as combined over a set of four LSs or a pair of RHAs in a hearing booth, as shown in the lower right corner.

### Simulation of Complex Acoustic Scenes

Binaural room simulation is based on HRTFs allowing for a spatialization of multiple VSSs, which can be efficiently computed on typical modern processors, if nothing other than direct propagation paths of the VSSs have to be calculated (Tsingos, Gallo, & Drettakis, 2004), as for example, in free field situations. If room acoustics need to be simulated, a high number of reflections have to be additionally spatialized, which is achieved by synthesizing BRIRs. However, even for models relying on geometrical acoustics, real-time simulation and synthesis of BRIRs are computationally challenging (Savioja & Svensson, 2015). For the auralization system, the simulation module Room Acoustics for Virtual Environments^2^ (RAVEN) was applied for BRIR calculation. RAVEN’s simulation models, which combine an image source method for DS and early reflections (Allen & Berkley, 1979), and a ray-tracing algorithm for reverberation (Krokstad, Strom, & Sørsdal, 1968) in a hybrid approach (Schröder, 2011), were adjusted to meet low-latency and processing requirements of the system. The software contains established and validated simulation algorithms (Pelzer, Aretz, & Vorländer, 2011), implemented as C++ libraries for the generation of BRIRs. The three parts of the BRIRs, that is, DS, early reflections, and reverberation tail, can be calculated separately.

Within the scope of the proposed system, these existing software implementations were applied and adjusted for applications in auditory experiments. While BRIRs are simulated with respect to the entrance of open or blocked ear canals, additional spatial filters (HARRIRs) are included referring to the positions of the HAs’ microphones. As a result, filter synthesis processes have to be extended to user-defined channel counts, requiring filters with four channels in the current system implementation. For BRIR and HARRIR calculation, a distinction between propagation simulation and filter synthesis is made. The propagation simulation includes the calculation of arrival times and levels of incoming sound waves for DS and reflections, while the filter synthesis is responsible for combining simulation results with directional characteristics of the receiver, that is, the virtual listener in the scene, and VSSs.

Since interactivity and variability of scenarios in auditory experiments are often limited to head rotations and only some translations (Lentz, Schröder, Vorländer, & Assenmacher, 2007), different configuration possibilities are proposed, varying in computational workload and simulation accuracy. To reduce the number of computations, perceptually less relevant simulation parts can be calculated before or during program initialization rather than during run-time. Table 2 shows four configurations which are considered common cases when realizing auditory experiments. For filter generation, the presented configurations always apply to the generation of both BRIRs and HARRIRs. The computational effort increases from Configuration A to Configuration D. Depending on the desired accuracy, available computational power and virtual scene, Configuration A might be preferred, although only DS updates are provided in real time. While dynamic binaural synthesis of DS is important (Laitinen, Pihlajamäki, Lösler, & Pulkki, 2012; Lindau, 2009), directions of incoming reflections can only be perceived up to the perceptual mixing time of BRIRs (Lindau, Kosanke, & Weinzierl, 2012). In a scenario where the test subject is sitting still, listening to static VSSs, rendering of the acoustic scene according to Configuration A or B is sufficiently accurate. However, given increased computational power, even on typical desktop computers, full room acoustic simulations for multiple static or moving sound sources can be applied in future auditory experiments. The section Benchmark Analysis of the Acoustic Simulation presents possible update rates for a restaurant scenario including updates of all BRIR and HARRIR parts, thus corresponding to Configuration D.

**Table 2.** Configurations for Room Acoustic Simulations and Filter Synthesis.

Configuration	Direct sound	Early reflections	Late reverberation
A	Real-time updates	Precalculated BRIRs or HARRIRs	Precalculated BRIRs or HARRIRs
B	Real-time updates	Real-time filter updates, precalculated image sources	Precalculated BRIRs or HARRIRs
C	Real-time updates	Real-time image source calculation and filter updates	Precalculated BRIRs or HARRIRs
D	Full real-time room acoustic simulation and filter updates		

*Note*. BRIRs = room impulse responses; HARRIRs = hearing aid-related room impulse responses.

Different parts of the binaural room impulse response (BRIR) and hearing aid-related room impulse response (HARRIR) filter sets are either calculated in real time or based on precalculated databases.

Different scenes for auditory experiments like classroom or restaurant situations can be created using 3D computer-aided design software, such as SketchUp (Timble Inc., Sunnyvale, California, United States). Acoustic characteristics of wall materials are adjusted to model room acoustic conditions, for example, by setting specified target reverberation times (cf. Pausch et al., 2016).

Configuration and management of the scene and its VSSs (Wefers & Vorländer, 2018), as well as convolution of simulated BRIRs and HARRIRs with the corresponding anechoic source signals, are carried out by the real-time auralization framework Virtual Acoustics (VA) (ITA Aachen, 2018; Wefers, 2015). Separated into modules, this environment allows rendering of VSSs, using various configurations like rendering based on DS only, including Doppler shifts (Strauss, 1998) in the case of moving VSSs (Wefers & Vorländer, 2015).

User movements are captured by an optical motion tracking system, consisting of four cameras (Flex13, NaturalPoint, Inc. DBA OptiTrack, Corvallis, Oregon) which operate at frame rates up to 120 Hz and feed the tracking signals back to the HAA module to trigger simulation updates.

### Processing of HA Signals

As outlined in the previous section, the filter generation module in RAVEN was extended to process HARTFs with user-defined channel count to create HARRIRs for a given virtual scene. An MHA is integrated into the procedure for full control over signal processing, including the HA fitting, as well as to insert convolved HA-related signals directly into the HA processing chain. The main purpose of an MHA is to compare and investigate different configurations and fittings for an HA device. For the proposed environment, the software Master Hearing Aid (HörTech gGmbH, Oldenburg, Germany) was selected. It supports low-latency, block-based signal processing, including basic signal processing algorithms (e.g., filter banks) among HA-related specific algorithms (e.g., dynamic range signal processing), which can be configured and selected as separate plug-ins and interconnected via a plug-in chain (Grimm et al., 2006). Apart from scripting possibilities, a graphical user interface is available for MATLAB (The MathWorks, Inc., Natick, Massachusetts, United States) which enables easy configuration of the MHA. Different fitting procedures can be configured by an audiologist and then selected by the supervisor of the experiment.

The two output signals of the MHA are reproduced by the left and right receiver of the RHAs. With this approach, no microphone signal needs to be processed simultaneously by the MHA, corresponding to perfect feedback cancellation. To make the system comparable to real HA devices, including their feedback problems, the signal path from the RHA’s receiver to the microphones can be simulated by convolving the two MHA output signals (cf. Figure 2) with the complex transfer function of the feedback path and by rerouting it back to the MHA input via software loopback. For this, an additional convolution for each RHA input channel would have to be realized. The output signals of these convolutions would then be added to the corresponding MHA input channels. This method is, however, not currently considered for the actual implementation.

### Combined Binaural Reproduction

#### HA-based reproduction

For full signal processing control, it is important that the RHAs provide access to raw microphone signals during HARTF measurements and raw HA receiver signals, for unprocessed playback. Therefore, a pair of custom-made, BTE receiver-in-the-ear RHAs (GN ReSound, Ballerup, Denmark), without digital signal processor but with full access to raw microphone and receiver signals, are used for playing back the MHA output signals. For measurement purposes, the RHAs are equipped with two omnidirectional micro-electro-mechanical systems microphones (Knowles, Itasca, Illinois, United States), which are installed at a distance of 6.2 mm and 1.7 mm below the enclosure surface. Signal playback is realized via a miniature magnetic receiver. To only minimally restrict user movement in auditory experiments, the signals are sent through slim cables with a diameter of 1.4 mm (Hi-Pro cable, Sonion, Roskilde, Denmark).

The subject’s perception of the external sound field works best either using an open fitting with a silicone dome or a tulip ear piece (Fretz, Stypulkowski, & Woods, 2001), provided this fitting strategy is suitable for the individual HA user. This fitting type is typically used together with BTE receiver-in-the-ear devices (Dillon, 2012) and will result in a reduced occlusion effect.

#### External sound field reproduction

High-end near-field monitors (K&H, O-110 Active Studio Monitor; Georg Neumann GmbH, Berlin, Germany) with a stable directivity pattern in a wide angular range (Georg Neumann GmbH, 2018), arranged according to the requirements given earlier, were selected to ensure high fidelity of the reproduced external sound field. Binaural LS reproduction is based on latest research results in the field of acoustic CTC systems (Lentz et al., 2007; Parodi & Rubak, 2010; Masiero, 2012).

For increased system stability (Lentz, 2008), the LS setup is extended to four LSs which are arranged in azimuth steps of ϕ=n·45deg, with *n* = 1, 3, 5, 7, sharing a zenith angle of ϑ=70deg and a listening distance of *r* ≈ 1.2 m with respect to the center of the listener’s interaural ear axis. As filtering technique, an *N*-CTC system formulation with *N* = 4 simultaneously playing LSs is used. The CTC matrix, containing inverted spatial transfer functions from each LS to the entrance of ear canals, that is, the playback HRTFs, is optimal in the least-squares sense using Tikhonov regularization with a regularization parameter of, for example, β = 0.05. For details about the implementation, the reader is referred to Masiero (2012).

#### Listening environment

As listening environment an acoustically optimized hearing booth (A:BOX, hearing test booth; Desone Modulare Akustik, Ingenieurgesellschaft mbH, Berlin, Germany) fulfilling ISO 8253-1 (2010), ISO 8253-2 (2009), and ISO 8253-3 (2012) with dimensions 2.3 × 2.3 × 1.98 m^3^ (Length × Width × Height) and a room volume of approximately *V* = 10.5 m^3^ was installed at the Institute of Technical Acoustics, RWTH Aachen University.

### System Latency

As the system’s workload varies considerably, depending on the selected configuration, see Table 2, simulation parameters and the number of VSSs, it is impossible to characterize the system’s latency by one EEL value alone. Owing to the separation of simulation components, user actions lead to simulation updates at different rates, resulting in individual latency values for each configuration and simulation component. The real-time auralization engine VA (ITA Aachen, 2018) was designed to process direct path updates based on user interactions in the next block of the audio buffer. Thus, minimum possible latency is determined by the selected audio buffer size. For the implemented system, a USB sound card (RME Fireface UC, Audio AG, Haimhausen, Germany) with Audio Stream Input/Output (ASIO) driver protocol was chosen. The buffer size of the sound card was set to either 128 or 256 samples, depending on available processing capacities, at a sampling rate of 44.1 kHz. Thus, the system provides a first reaction, usually the binaural synthesis of the VSS’s DS, with a delay of at least one buffer. The total EEL is, however, also affected by latencies introduced by the motion-tracking system (Friston & Steed, 2014; Steed, 2008) and the sound card’s DA conversion speed.

### Discussion

This subsection briefly discusses to what extent requirements not related to experimental investigations are fulfilled by the system implementation. The measurement and quality of spatial transfer functions, a benchmark of room acoustic simulation, combined binaural reproduction, and system latency are evaluated in experiments which are discussed in the upcoming sections.

As the implemented real-time auralization system had already successfully been applied in initial experiments for auditory research on subjects with HL (Pausch et al., 2016), the overall system design can be considered successful. The simulation environment created allows for generating complex virtual scenes based on geometrical acoustics. According to user specifications, VSSs and receivers can be placed anywhere in the designed virtual room with arbitrary orientation. Directional characteristics of VSSs and the receiver can be set according to directivity databases and generic or individually measured spatial transfer function databases, containing HRTF and HARTF data sets. An interface of the simulation environment allows for an easy creation of virtual scenes, using SketchUp, and thus provides a user-friendly method of scene definition and modification.

The real-time auralization engine facilitates continuous and artifact-free audio streams of spatialized VSSs, while accounting for user interaction tracked by a wireless optical-motion tracking system. To ensure real-time reproduction, the number of VSSs and the geometrical complexity of the scene are, however, limited. For an entire real-time simulation of BRIRs (cf. Configuration D in Table 2), including numerous reflections, the required processing power exceeds capacities of standard desktop PCs. As a result, insufficient update rates can cause audible artifacts, especially if more than one VSS is auralized in a dynamic virtual scene. Thus, depending on the scene’s complexity, the simulation configuration and its parameters have to be individually adjusted. A MATLAB interface enables the implementation of scripts for scene modification and parameter refinement. It also supports integration into experimental procedures and time-critical paradigms.

The challenge of integrating HAs was solved by utilizing carefully designed custom-made RHAs with access to raw microphone and receiver signals, thus fulfilling the requirements of full signal control. All HA algorithms involved can be fully controlled through the use of a powerful MHA real-time software platform, remedying a potential bias relating to unpredictable behavior of proprietary HA algorithms. The MHA also provides a MATLAB interface which facilitates control and preparation of experiments.

## Experimental Methods

### Measurement of Spatial Transfer Functions

The following section describes measurement and analysis of HRTF and HARTF data sets, obtained from an artificial head with simplified torso and detailed ear geometry (Schmitz, 1995).

The measurements cover directions on a sphere with a radius of 1.86 m, relating to the center of the artificial head’s interaural axis, sampled on an equiangular grid with 1deg×1deg in azimuth and zenith angles. The artificial head was placed on a turntable to measure all azimuth rotations at the given resolution. As the remote-controlled arm with mounted measurement LS can only measure zenith angles between 0° and 120° on account of practical restrictions, two sequential measurement cycles were carried out. In the first one, the upper hemisphere up to ϑ=95deg was measured to provide sufficient overlap with the horizontal plane, while in the second one, the artificial head was mounted upside down to cover the lower hemisphere from ϑ=180deg to 85deg. Before each measurement cycle, the artificial head was set to a viewing direction of 0deg azimuth by zeroing the interaural time difference (ITD; Katz & Noisternig, 2014). Results of both measurement cycles were subsequently combined. All measurements were carried out in a hemi-anechoic chamber, with dimensions 11 × 5.97 × 4.5 m^3^ (Length × Width × Height) and a room volume of 295.5 m^3^, featuring a lower frequency limit of approximately 100 Hz.

As excitation signal, an exponentially swept sine between 20 and 20000 Hz was used with a length of 2^15^ samples at a sampling frequency of 44.1 kHz. The digital-to-analog-converted measurement signal (RME Hammerfall DSP Multiface II, Audio AG, Haimhausen, Germany) was played back through a custom-made broadband LS, equipped with a 2-in. driver (OmnesAudio BB2.01, Blue Planet Acoustic, Frankfurt, Germany), and a frequency range of 200 Hz to 20 kHz. After amplification and analog-to-digital (A/D) conversion (RME Octamic / Multiface II, Audio AG, Haimhausen, Germany), the sweep response was recorded by the artificial head’s microphones (Schoeps CCM 2H, Schoeps GmbH, Karlsruhe, Germany), and the microphones of the two RHAs mounted on the artificial head. Each channel of the input measurement chain was calibrated, using a defined voltage source, to avoid mismatched channel gains.

To preserve useful time segments of head-related impulse responses (HRIRs) and hearing aid-related impulse responses (HARIRs^3^), each data set was time shifted by the global minimum onset delay. Shifted data sets were subsequently cropped to a length of 256 samples, and the right side of a Hann window with a length of 89 samples was applied for fading out the impulse response. As reference measurement, that is, without the presence of the artificial head, a free-field microphone (Type 4190, Brüel & Kjær, Nærum, Denmark) was used with measurement amplifier (Type 2606, Brüel & Kjær, Nærum, Denmark) and A/D converter (RME Fireface UC, Audio AG, Haimhausen, Germany) to spectrally divide measured HARTFs in complex frequency domain, thus containing the HA microphone transfer function. The HRTF data set was spectrally divided by transfer functions measured between LS and respective microphone of the artificial head.

To investigate basic differences between the two spatial transfer function data sets, direction- and frequency-dependent interaural level differences (ILDs) were evaluated for a subset of azimuth angles, namely, ϕ=k·30, with k={0,1,…,5}, in the horizontal plane by dividing the complex spectrum of signals on the right-ear side by the ones on the left, subsequently calculating the magnitude spectra in dB. Another binaural cue of interest is ITD, which was calculated following the interaural cross correlation coefficient method, proposed by Katz and Noisternig (2014), in a frequency range between 100 and 1500 Hz.

### Benchmark Analysis of the Acoustic Simulation

For selecting an adequate configuration for room acoustic simulation to be used in interactive experiments, it becomes important to investigate computational demands of the selected configuration. This section, therefore, presents an evaluation of a virtual restaurant scene with multiple VSSs.

The simulation library allows for separate simulation of different parts of the BRIR or HARRIR, see Table 2. For DS, an audibility test checks for VSS obstructions by objects or walls with respect to the virtual receiver’s position. This test has to be updated whenever the receiver or the VSS moves translationally. The calculation of early reflections is split into the generation of image sources and audibility test. Whenever the VSS changes its position, new image sources have to be generated, followed by audibility tests, checking the validity of reflection paths. In the case of a translational receiver movement, only the audibility test is executed (Vorländer, 2007).

Reverberation is simulated by a ray-tracing algorithm. Because of frequency-dependent absorption and scattering, it is executed for the full audible bandwidth covering ten octave bands with center frequencies between 31.5 Hz and 16 kHz. The output of this algorithm comprises energy decay histograms for these ten octave bands, with a minimum length of the room’s longest estimated reverberation time.

#### Acoustic scene

**Figure 3.**
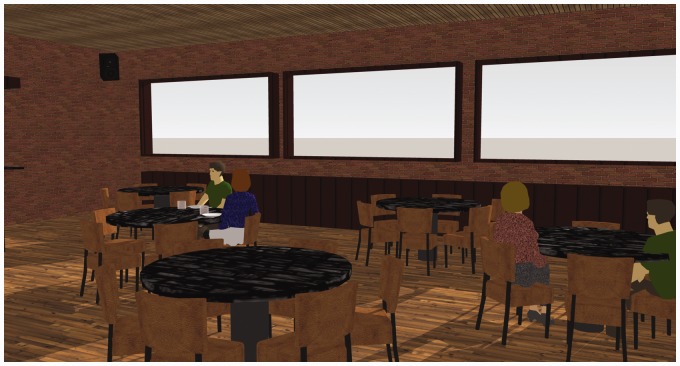
Visual representation of the complex restaurant scene used for the simulation benchmark analysis. The camera view corresponds to the virtual receiver’s position and orientation.

A restaurant scenario with three VSSs was selected as example scene for the acoustic simulation benchmark, Figure 3 showing a visual representation thereof. Representing a complex but controlled scenario, such a scene is likely to occur in everyday life of subjects with NH or HL. For the latter, this is a challenging situation, as speech intelligibility is reduced due to various distracting sound sources and unfavorable room acoustic conditions.

**Figure 4.**
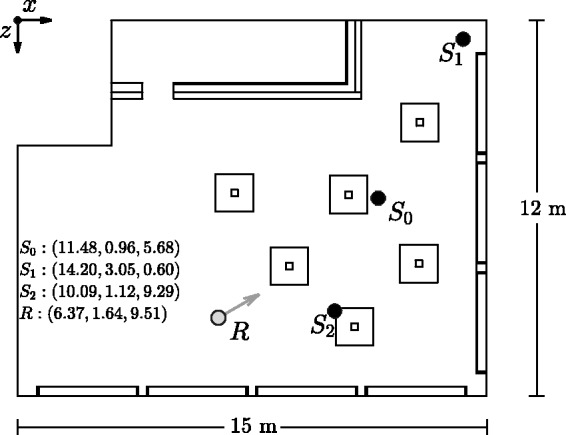
Top view of the complex restaurant scene used for the simulation benchmark analysis. The scene contains three omnidirectional virtual sound sources, *S*_0_, *S*_1_, and *S*_2_, and a virtual receiver *R* with orientation as shown by the gray arrow. The respective positions are specified as three-dimensional Cartesian coordinates (*x*, *y*, *z*), given in meters.

A top view of the acoustic room model is shown in Figure 4, including positions of the VSSs and the virtual receiver’s position and orientation. The underlying model consists of 387 polygons and 109 surfaces with a volume of 581 m^3^ and a surface area of 625 m^2^. During room acoustic simulations, all VSSs were modeled as omnidirectional sources, making the simulation independent of source signals. Example source signals for *S*_0_ and *S*_2_ can be cutlery noise or a talking person. The VSS *S*_1_, located in the top corner of the room, represents, for example, a LS reproduction of a music signal.

For the defined receiver position, the room has a mean reverberation time *T*_30_ = 0.89 s, averaged over 500 Hz and 1000 Hz octave bands. The perceptual mixing time of the room is estimated by

(1) $$t_{\text{mp}95}=0.0117\frac{\text{ms}}{{\text{m}}^{3}}\cdot 581{\text{m}}^{3}+50.1\text{ms}\approx 56.9\text{ms},$$

applying the model-based predictor (Lindau et al., 2012). This time determines the required simulation length for reverberation updates.

#### Benchmark procedure

The benchmark evaluation is split into two parts: room acoustic simulation, identical to a conventional simulation for subjects with NH, and the filter synthesis generating binaural filters as well as filters for the input channels of the RHAs. To guarantee an accurate measurement of calculation times, each simulation part was evaluated separately. This allows for the comparison of computational demands for simulation tasks, whereas in the implementation of the full auralization system, multiple threads are running in parallel at different priorities, making it difficult to measure calculation times.

Binaural filters (two output channels) and HA-related filters (four output channels) were processed in one function and thus evaluated collectively in one six-channel filter synthesis. The total filter length of BRIRs and HARRIRs was set to 2,000 ms. In an optimized configuration, energy decay and reverberation filters were only simulated for the first 200 ms, which is well above perceptual mixing time, and were then extrapolated for the remaining part.

The benchmark analysis was carried out using a computer with an Intel Core i7-3770 CPU @ 3.40 GHz running a 64-bit Windows 7 Enterprise operating system. All simulation and filter synthesis tasks were executed and measured ten times. For all three VSSs, image sources were calculated up to the second order, ray-tracing was set to 3,000 particles per octave band, with diffuse rain technique (Schröder, 2011) enabled. For calculating maximum update rates, the calculation times for all three VSSs were first summed up, as they are processed sequentially in the current implementation, and then weighted by a factor $f=\frac{1}{3}$, which accounts for other tasks running simultaneously. Only one third of the CPU time is assigned to the simulation engine, which cannot be accurately controlled in the final application owing to inconsistent workload and multithreading processing. Application tests and other investigations have confirmed this estimation (Aspöck et al., 2014).

### Combined Binaural Reproduction

#### HA-based reproduction

For measuring the receiver transfer function, the RHAs were mounted on an artificial head (HMS III digital, HEAD Acoustics, Herzogenrath, Germany) with ear simulator, meeting the specifications of ITU-T P.57 (2009). The ear simulator’s output signal was amplified and recorded, using a charge amplifier (Type 2692A, Nexus, Brüel & Kjær, Nærum, Denmark) connected to an audio interface (RME Fireface UC, Audio AG, Haimhausen, Germany). An exponentially swept sine between 50 and 20000 Hz, at a sampling frequency of 44.1 kHz, with a length of 2^18^ samples, was used to obtain RHA receiver transfer functions. Two different ear pieces were measured: a silicone dome with holes and a tulip ear piece. Resulting impulse responses were cropped to a length of 2^15^ samples. After smoothing the obtained complex transfer functions using 1/6-octave band filters (Hatziantoniou & Mourjopoulos, 2000), magnitude spectra were calculated in dB. Measurements were repeated 10 times, repositioning the respective ear piece to account for measurement uncertainty.

With reference to different types of ear pieces, it is of particular interest how perception of the external sound field is altered when wearing RHAs. Therefore, the frequency-dependent damping of the two ear pieces was measured as an example for one source position. For measuring, an LS was placed at an azimuth angle of ϕ=45deg in the horizontal plane, at a distance of 1.2 m, the RHA being attached to the left-ear side of the same artificial head (HMS III digital, HEAD Acoustics, Herzogenrath, Germany). An exponentially swept sine, at a sampling frequency of 44.1 kHz with a length of 2^18^ samples, was used to obtain spatial transfer functions in two sequential measurements, with and without attached RHA per ear piece type. Impulse responses were windowed and cropped, using the right side of a Hann window, applied to between 265 and 354 samples. Subsequently, we divided the complex transfer functions with attached RHAs by the transfer functions without attached RHAs and calculated the magnitude spectra in dB to obtain relative spectral attenuations caused by the respective ear piece type.

#### External sound field reproduction

Performance of CTC systems can be quantified analyzing their frequency-dependent CS when utilizing the CTC matrix on playback transfer functions (Gardner, 1998). Following the definitions by Akeroyd et al. (2007) and Majdak et al. (2013), positive values represent higher CS, indicating that the perceived reproduced binaural signal will be closer to the original binaural input signal (Parodi & Rubak, 2011).

To analyze the system performance in the case of user rotation, the rotation-dependent CS was calculated based on measurements in a nonideal listening environment with room reflections. For these measurements, an artificial head with simplified torso and detailed ear geometry (Schmitz, 1995) was placed in the center of the hearing booth, on a turntable, which was rotated in azimuth steps of 10deg to sequentially measure BRIRs. The artificial head was set to an ear height of 1.15 m. An exponentially swept sine at a sampling frequency of 48 kHz between 20 and 20000 Hz with a length of 2^16^ samples was used as excitation signal, sequentially driving four LSs (K&H, O-110 Active Studio Monitor; Georg Neumann GmbH, Berlin, Germany). In this measurement scenario, the LSs were placed at azimuth angles of ϕ={40deg,140deg,220deg,320deg}, sharing a zenith angle of θ=70deg and a listening distance of 1.2 m with respect to the center of the artificial head’s interaural axis.

The beginning of measured BRIRs was windowed by applying the left side of a Hann window with a length of 45 samples. The right side of a Hann window, with a length of 221 samples, starting 44,100 samples after the onset of the impulse response (ISO 3382-1, 2009), was used for fade out without additional cropping. Corresponding HRTFs were obtained from windowed BRIRs by applying the right side of a Hann window with a length of 89 samples, starting 14 samples after the impulse response onset. These windowed HRTF data sets were additionally cropped to obtain a length of 256 samples.

To account for LS transducer characteristics, measured HRTF and BRIR data sets were convolved in a circular manner with the inverted LS on-axis free field responses, which were realized as minimum-phase filters with a length of 256 samples. These postprocessed HRTFs and BRIRs, respectively, constitute ideal and practical versions of the playback HRTF matrix. Postprocessed HRTFs were used for calculating the CTC matrix. Under these conditions, both the ideal and the practically achieved CS, room reflections included, were calculated.

#### Listening environment

To objectively quantify the example listening environment, room acoustic measurements were taken with two source and six receiver positions (precision level). Normative demands of ISO 3382-2 (2008) with reference to measurement positions were not met due to space restrictions. Because of room size limitations and sound field quality in the hearing booth, results should be interpreted carefully. For taking measurements, a 1/2″ random incidence microphone (Type 4134, Brüel & Kjær, Nærum, Denmark) was used, together with a charge amplifier (Type 2692A, Nexus, Brüel & Kjær, Nærum, Denmark) and an audio interface (RME Fireface UC, Audio AG, Haimhausen, Germany). An exponentially swept sine, with a length of 2^16^ samples at a sampling frequency of 48 kHz, covering a frequency range of 20 to 20000 Hz, was bandpass-filtered and matched energetically by a digital loudspeaker management system (FourAudio, Herzogenrath, Germany), adhering to the respective audio crossover specifications and directivity measurements of the omnidirectional measurement LS (Behler & Müller, 2000). The measurement signal was amplified using a custom-made class B power amplifier and played back through the measurement LS, with two averages per source-receiver combination. Reverberation times *T*_30_ were calculated using the ITA-Toolbox (Berzborn, Bomhardt, Klein, Richter, & Vorländer, 2017), with applied noise detection and compensation according to Lundeby, Vigran, Bietz, and Vorländer (1995). Mean results were obtained by arithmetically averaging respective parameter results per octave band.

BNLs were measured five times for 12 s at the listening position, that is, in the center of the hearing booth at a height of 1.2 m. For this purpose, a 1/2″ low-noise measurement microphone (Type 40HL, GRAS Sound & Vibration A/S, Holte, Denmark) was used in combination with a charge amplifier (Type 2692A, Nexus, Brüel & Kjær, Nærum, Denmark) and an audio interface (RME Fireface UC, Audio AG, Haimhausen, Germany). Mean BNLs were obtained by energetic averaging respective octave-band levels across measurements.

### Combined System Latency

The procedure for measuring EEL is based on measuring absolute times of arrival of impulse responses, involving rendering and reproduction delays. To the best knowledge of the authors, no publication exists reporting the latency of the tracking system used (Flex 13; Motive 1.8.0 Final; NaturalPoint, Inc. DBA OptiTrack, Corvallis, Oregon). As such a measurement is beyond the scope of this article, only static EEL was measured for the implemented system. This was done by placing an artificial head (Schmitz, 1995) in the center of a hearing booth at an ear height of 1.2 m. The artificial head was equipped with the RHAs and a head-mounted rigid body for optical tracking systems, enabling the determination of head position and orientation. For correct auralization, the offset of the head-mounted rigid body was adjusted with respect to the center of the interaural axis by adding a displacement to the rigid body’s geometric center.

**Figure 5.**
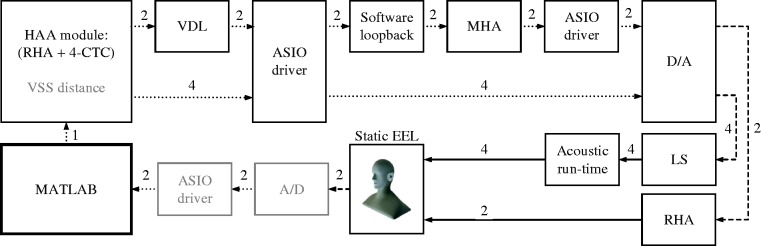
Flow diagram to measure the system’s static EEL. An exponential sweep was generated in MATLAB and played back by a VSS at a distance of 2 m in front of a virtual listener in a virtual room. In the HAA module, auralization filters were utilized from precalculated databases to generate signals for the HA-based and LS-based path. The HA-based signal is delayed using a VDL, looped back to be processed in the MHA, sent through a D/A converter, and finally played back via RHAs. The LS-based signals are processed by the 4-CTC filter network, D/A converted, and sent to four LSs. Sweep responses were measured by the artificial head’s microphones and deconvolved in MATLAB to obtain impulse responses for the respective simulation path. Dotted and dashed black lines represent signals in digital and analog domain, respectively, whereas solid black lines represent acoustic signals. VSS distance and latencies introduced by the A/D converter and the ASIO driver block, all in gray, were not included in the final static EEL. *Note.* HAA = hearing aid auralization; RHA = research hearing aid; CTC = crosstalk cancellation; VDL = variable delay line; ASIO = audio stream input/output; MHA = master hearing aid; EEL = end-to-end latency; A/D = analog-to-digital; LS = loudspeaker; D/A = digital-to-analog; VSS = virtual sound source.

Figure 5 shows the signal flow and all components involved contributing to the final static EEL. A VSS was placed in front of the virtual listener, that is, at 0deg azimuth in the horizontal plane, at a distance of 2 m in a virtual room. The VSS plays back an exponentially swept sine with a length of 2^16^ samples at a sampling frequency of 44.1 kHz in a frequency range of 20 to 20000 Hz, generated in MATLAB. Room acoustic filters, that is, BRIRs and HARRIRs, were synthesized based on HARTF and HRTF data sets in the HAA module, running on a desktop PC (Intel Core i7-4770 @ 3.4 GHz, Windows 7 Enterprise). The signal for the RHA-based playback was additionally time delayed in the software module using the VDL to obtain a relative delay of 221 samples, corresponding to 5 ms between the RHA and the external sound field reproduction, accounting for real-life HA delays (Stone et al., 2008). This signal was then looped back through the audio interface’s software loopback (RME Fireface UC, TotalMix, Audio AG, Haimhausen, Germany) and used as input for the MHA software (Grimm et al., 2006). The plug-in chain of the MHA comprised stages for downsampling (Factor 2, plug-in downsample), calibration (plug-in splcalib) which calls sub-plug-ins such as a fast Fourier transform filterbank, limiter, compressor, and an overlap-add plug-in for resynthesis. After upsampling (Factor 2, plug-in upsample), the signals were played back through the RHAs. Sweep responses of both binaural playback paths were measured by the artificial head’s microphones, sent back to the audio interface, and deconvolved using MATLAB and the ITA-Toolbox (Berzborn et al., 2017), yielding an impulse response for HA-based as well as LS-based auralization paths. Note that VSS distance and latencies, due to A/D and ASIO driver, are displayed in gray, as they are not included when calculating static EELs. A list of relevant setup parameters is provided in Table 3.

**Table 3.** Parameter Settings of the Extended Binaural Real-Time Auralization System, as Used for Static End-To-End Latency (EEL) Measurements.

RME Fireface UC	
Sampling rate (Hz)	44100
Buffer size (samples)	128

Measurement signal	
Frequency range (Hz)	20–20000
Length (samples)	2^16^

HAA module	
Number of channels	6
HRTF filter length (samples)	256
HARTF filter length (samples)	256
BRIR filter length (samples)	44,100
HARRIR filter length (samples)	44,100
CTC filter length (samples)	1,226
Regularization parameter β(·)	0.01

MHA	
Number of channels	2
Sampling rate (Hz)	22050 (downsampled)
Fragment size (samples)	256
Plug-in chain	(downsample…
	splcalib upsample)

*Note.* HAA = hearing aid auralization; HRTF = head-related transfer function; HARTF = hearing aid-related transfer function; BRIR = room impulse response; HARRIR = hearing aid-related room impulse response; CTC = crosstalk cancellation; MHA = master hearing aid.

Settings related to the audio interface used (RME Fireface UC), measurement signal, and hearing aid auralization (HAA) module. Settings of the master hearing aid (MHA) only cover modifications applied to the provided standard configuration file mha_hearingaid.cfg.

## Experimental Results

### Measurement of Spatial Transfer Functions

**Figure 6.**
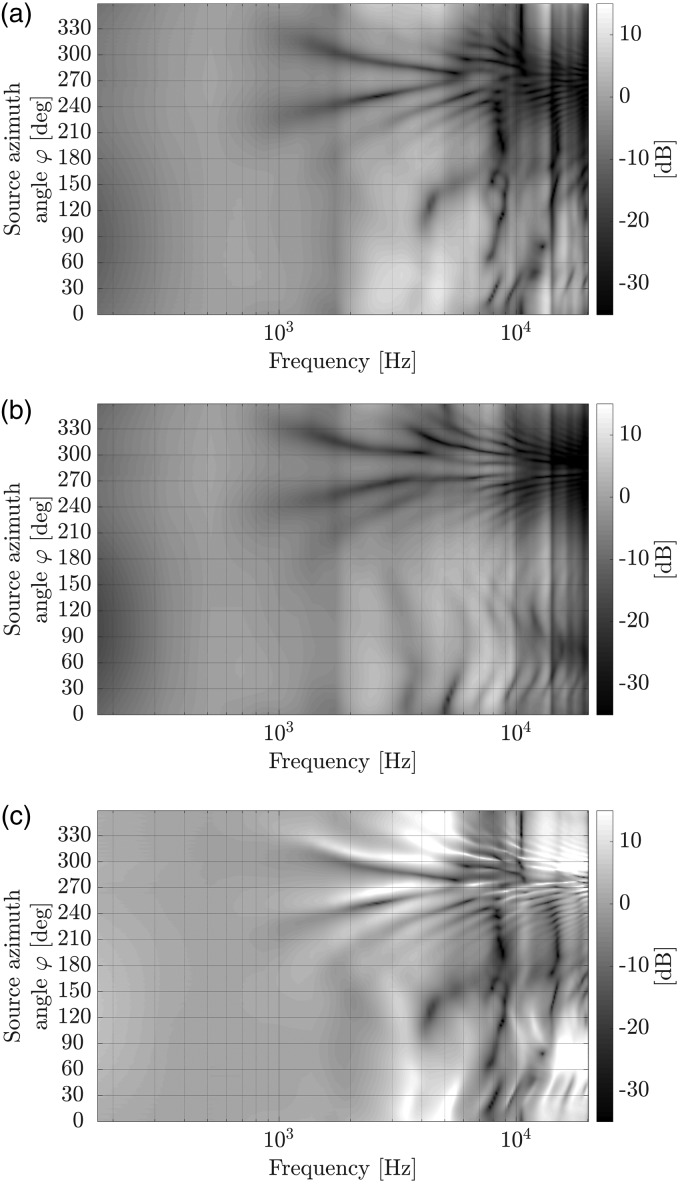
Magnitude spectra of generic spatial transfer functions for varying source azimuth angles in the horizontal plane, measured by the left ear microphone of an artificial head and the left research hearing aid’s front microphone. (a) Head-related transfer function (HRTF) data set. (b) Hearing aid-related transfer function (HARTF) data set. (c) Spectral difference between the HRTF and HARTF data set.

In Figure 6(a) and (b), magnitude spectra of both measured generic HRTF and HARTF data sets are plotted in dB for all measured source azimuth angles in the horizontal plane. To highlight the different quality of both data sets, the spectral difference obtained by dividing the complex spectrum of the HRTF by that of the HARTF and subsequently calculating the magnitude spectrum in dB is shown in Figure 6(c). Note that only the left microphone signal of the artificial head and the left RHA’s front microphone and their spectral magnitude differences are shown in the subfigures.

**Figure 7.**
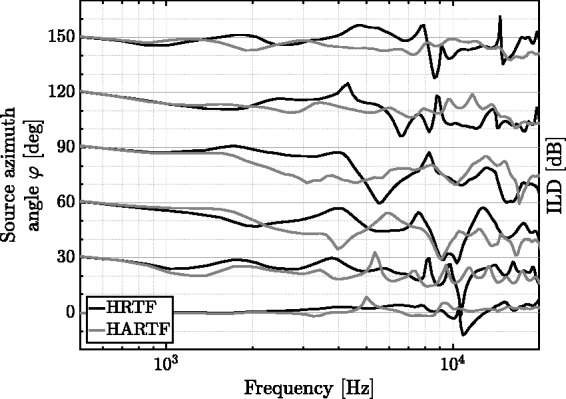
ILD of HRTF and HARTF data sets measured by the RHAs’ front microphones and evaluated for selected source azimuth angles ϕ in the horizontal plane. For the right ordinate axis, horizontal solid major grid lines represent an ILD of 0 dB for the respective source azimuth angle whereas distances between two horizontal minor grid lines correspond to 10 dB. *Note.* HRTF = head-related transfer function; HARTF = hearing aid-related transfer function; ILD = interaural level difference.

Figure 7 shows ILDs of both data sets for a subset of source azimuth angles in the horizontal plane on ipsilateral ear side. Major horizontal grid lines constitute an ILD of 0 dB for the respective source azimuth angle, whereas each horizontal minor grid line represents level changes of 10 dB. ILDs of both data sets differ substantially, and spectral deviations between opposed microphone signals of the same data set type become visible above frequencies of around 500 Hz. For the selected subset of source azimuth angles, ILDs attain maximum values of –51.4 dB (HRTF) and –47.2 dB (HARTF).

**Figure 8.**
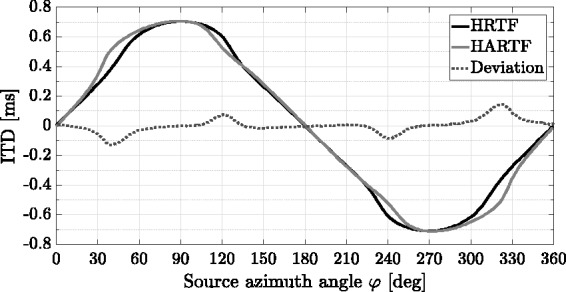
ITD of measured HRTF and HARTF data sets, evaluated for all source azimuth angles ϕ in the horizontal plane. The latter data set is based on front microphone signals of the research hearing aids. Deviation between ITD values of HRTF and HARTF data sets are plotted as dotted gray line. *Note.* HRTF = head-related transfer function; HARTF = hearing aid-related transfer function; ITD = interaural time difference.

Results for ITDs of both HRTF and HARTF data sets, and the deviation between the data sets in the horizontal plane, are shown in Figure 8. For the HARTF data set, only spatial transfer functions measured by front microphones of the RHAs were evaluated. Both ITD curves show sinusoidal shapes with maxima at 87deg (HRTF) and 83deg (HARTF), and minima at 264deg (HRTF) and 269deg (HARTF), while exhibiting a common peak amplitude of ±0.7 ms which corresponds to a maximum path difference of about 24.1 cm (given a speed of sound of 344 m/s). The largest ITD deviation between the two data sets can be observed at source azimuth angles of 19° to 73° and 293° to 355°, as well as at 107° to 133° and 226° to 259°, respectively.

### Benchmark analysis of the acoustic simulation

Table 4 shows the results of the benchmark analysis for five different room acoustic simulation tasks, averaged over ten calculation time measurements. The DS audibility check can be provided at high update rates, while update rates of early reflections for approximately 22 audible image sources per VSS are below 50 Hz. Calculation times for ray-tracing are considerably higher. If the energy decay is calculated separately for each VSS in accordance with the physical shape of the room, update rates of only 0.2 Hz can be achieved.

**Table 4.** Mean Calculation Times With Standard Deviations (*M* ± *SD*) and Highest Possible Update Rates for Room Acoustic Simulations Separated into Simulation Models for Direct Sound, Early Reflections (Image Sources), and Late Reverberation (Ray-Tracing).

Simulation task	Virtual sound sources			Maximum update rate (Hz)
	*S*_0_	*S*_1_	*S*_2_	
	*M* ± *SD* (ms)	*M* ± *SD* (ms)	*M* ± *SD* (ms)	
Direct sound, audibility check	0.9e–3 ± 0.0e–3	3.2e–3 ± 0.2e–3	0.3e–3 ± 0.0e–3	75.6e3
Image sources, audibility check	9.9 ± 0.1	13.6 ± 1.0	12.3 ± 4.6	9.3
Image sources, full update	16.9 ± 1.5	16.5 ± 0.3	16.3 ± 0.2	6.7
Ray-tracing (2,000 ms)	595.6 ± 8.6	602.5 ± 1.6	577.9 ± 1.8	0.2
Ray-tracing (200 ms)	238.5 ± 1.5	242.8 ± 1.2	224.7 ± 0.9	0.5

Table 5 shows calculation times for the six-channel filter synthesis for all three room impulse response parts. A filter update, especially of DS, which dominates the listener’s perceptual impression, is required frequently, for example, in the case of user rotation. While the filter synthesis for DS and early reflections can be calculated for high update rates above 100 Hz, the full reverberation synthesis does not allow update rates higher than 1 Hz. The perceptual mixing time implementation, which only updates the filter up to 200 ms, increases possible update rates to an acceptable rate of 6 Hz, contrasted to the full length synthesis of the reverberation.

**Table 5.** Mean Calculation Times With Standard Deviations (*M* ± *SD*) and Highest Possible Update Rates for Filter Synthesis of Direct Sound, Early Reflections (Image Sources), and Late Reverberation (Ray-Tracing).

Filter synthesis task	Virtual sound sources			Maximum update rate (Hz)
	*S*_0_	*S*_1_	*S*_2_	
	*M* ± *SD* (ms)	*M* ± *SD* (ms)	*M* ± *SD* (ms)	
Direct sound	0.1 ± 0.0	0.1 ± 0.0	0.1 ± 0.0	923.7
Image sources	0.2 ± 0.0	0.2 ± 0.0	0.2 ± 0.0	545.6
Ray-tracing (2,000 ms)	196.3 ± 0.7	195.2 ± 1.1	196.1 ± 1.0	0.6
Ray-tracing (200 ms)	19.6 ± 0.1	19.6 ± 0.1	20.4 ± 0.3	5.6

*Note.* All filters are based on head-related transfer functions (HRTFs) and hearing aid-related transfer functions (HARTFs) with a length of 128 samples and a sampling rate of 44.1 kHz. Each filter was calculated for a total of six channels, two corresponding to the signals at the entrance of the subject’s ear canals, and four to the simulated microphone input signals for a pair of research hearing aids.

### Combined Binaural Reproduction

#### HA-based reproduction

**Figure 9.**
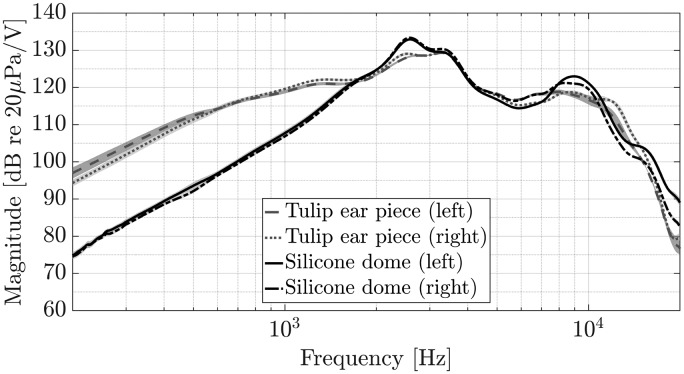
Magnitude spectra of left and right RHA receivers’ transfer functions with silicone dome or tulip ear piece measured by an ear simulator at −20 dBV input level. Magnitude spectra were smoothed using filters with constant relative bandwidth of one-sixth octave. Gray-shaded areas mark the 95% confidence interval of the mean. *Note.* RHA = research hearing aid.

Measurement results of the RHAs’ receiver transfer functions are shown in Figure 9. Regardless of the ear piece used, distinct energy peaks are observed at first and second ear simulator resonances, that is, around 3 and 9 kHz. In addition, left and right receiver responses are almost identical for the respective ear piece. Measurements conducted with the tulip ear piece feature better low-frequency response owing to less leakage, resulting in an increased pressure chamber effect (Dillon, 2012).

**Figure 10.**
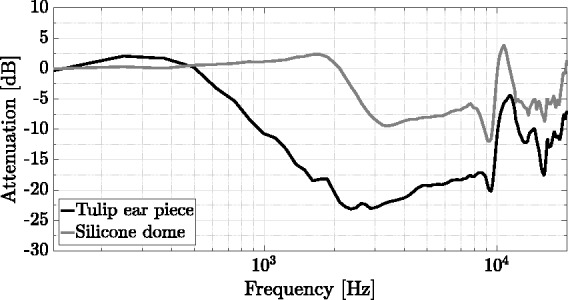
External sound field attenuation on ipsilateral side for a sound incidence angle of $\phi {=}^{45}\circ$ in the horizontal plane, depending on the ear piece type used together with the research hearing aids.

Measurement results related to the damping of external sound fields depending on ear piece type are shown in Figure 10. The tulip ear piece attenuates the external sound field above frequencies of 500 Hz by approximately −10 dB/octave up to 3 kHz, with maximum attenuation of −23 dB. Damping slightly decreases to a resonance frequency of 11 kHz, causing an attenuation drop, and fluctuates around −10 dB for higher frequencies. For the silicone dome, the lower cutoff frequency is higher compared with the tulip ear piece owing to increased leakage. Almost no influence on external sound field perception is observed up to frequencies of 2.3 kHz, before attenuation increases by −15 dB/octave up to 3.3 kHz, with a maximum value of approximately −10 dB. Similar to the tulip ear piece, attenuation slightly decreases, then drops considerably at a resonance frequency of 11 kHz before increasing again to about −5 dB for frequencies above.

#### External sound field reproduction

**Figure 11.**
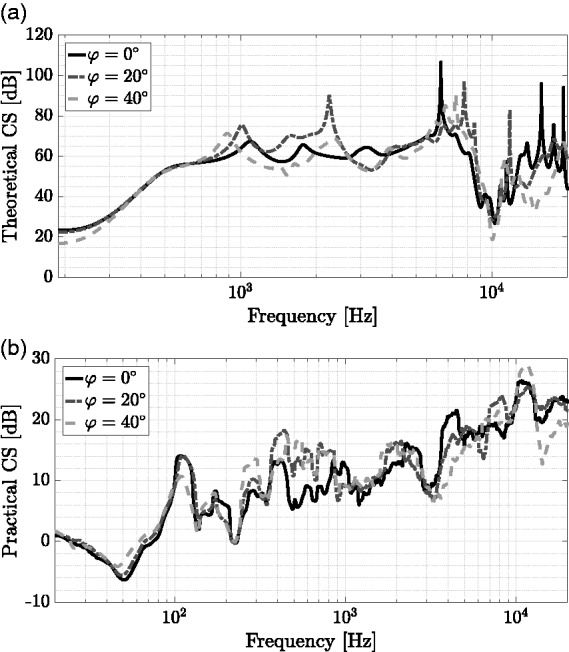
Rotation-dependent left-ear CS of the acoustic crosstalk cancellation (CTC) system, implemented as 4-CTC (β = 0.05). The CS was calculated (a) without and (b) with additional room reflections in spatial playback transfer functions, that is, head-related transfer functions (HRTFs, theoretical CS) or binaural room impulse responses (BRIRs, practical CS), both measured in the example listening environment. (a) Calculated CS after applying CTC filters on playback HRTFs. (b) Calculated CS after applying CTC filters on playback BRIRs. For better readability, spectral smoothing using filters with constant relative bandwidth of one-third octave was applied. *Note.* CS = channel separation.

The rotation-dependent left-ear CS is plotted over frequency in Figure 11. To facilitate readability, only a subset of artificial head rotations, that is, 0deg,20deg, and 40deg azimuth, was selected. Figure 11(a) shows the theoretical CS when applying CTC filters on playback HRTFs. The achieved CS lies within approximately 17 to 75 dB (up to peak values of 107 dB) for the selected head rotations, showing an increasing performance up to high frequencies with a drop between 8.5 and 11.5 kHz. Figure 11(b) shows practical CS when CTC filters are applied to playback BRIRs. In this case, the CS is notably lower, compared with an ideal case, because of the influence of room reflections. In the frequency range of about 25 to 70 Hz, the CS exhibits negative values. In general, an increasing performance trend toward higher frequencies can be observed. To obtain single-number ratings, average CS values were calculated for both scenarios and selected artificial head rotations, covering different frequency ranges, namely, broadband between 0.02 and 24 kHz, 0.3 and 2 kHz, and 4 and 16 kHz, as shown in Table 6.

**Table 6.** Mean Channel Separation (CS) Values With Standard Deviations (*M* ± *SD*), Using the Described Acoustic Crosstalk Cancellation (CTC) Filter Network.

Head rotation * ϕ* (°)	CS without room reflections			CS with room reflections		
	Broadband	0.3–2 kHz	4–16 kHz	Broadband	0.3–2 kHz	4–16 kHz
	*M* ± *SD* (dB)	*M* ± *SD* (dB)	*M* ± *SD* (dB)	*M* ± *SD* (dB)	*M* ± *SD* (dB)	*M* ± *SD* (dB)
0	52.9 ± 15.1	60.6 ± 3.4	56.3 ± 14.4	16.9 ± 7.3	7.8 ± 6.0	17.9 ± 6.6
20	54.9 ± 15.0	66.2 ± 6.0	55.4 ± 14.6	17.5 ± 7.3	9.9 ± 6.2	19.0 ± 6.6
40	51.5 ± 14.8	58.5 ± 4.9	52.4 ± 16.8	15.9 ± 7.8	9.9 ± 6.4	16.7 ± 7.8

*Note.* CS = channel separation.

Values were calculated on the basis of artificial head measurements of playback head-related transfer function (HRTF) data sets for different head rotations *ϕ*, conducted in the example listening environment. CS values were calculated for different frequency ranges, namely, broadband between 0.02 and 24 kHz, 0.3 and 2 kHz, and 4 and 16 kHz, by applying CTC filters either to playback HRTF or binaural room impulse response (BRIR) data sets, thus, either containing or not containing additional room reflections.

#### Listening environment

**Figure 12.**
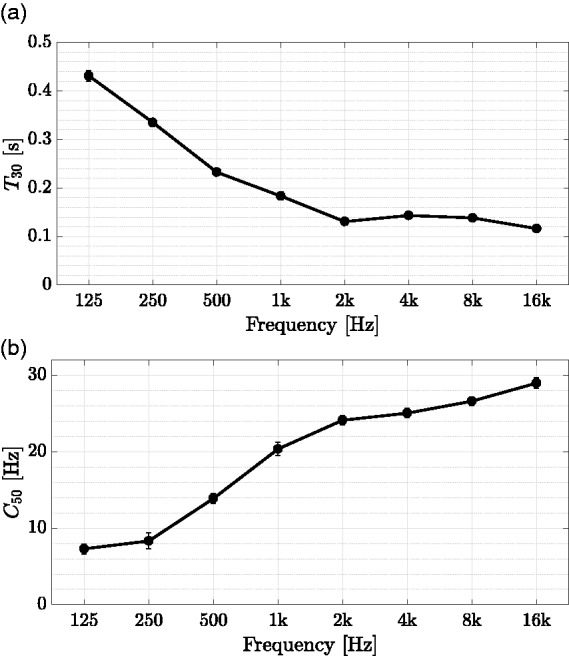
Selected room acoustic parameters of the example listening environment. Error bars indicate 95% confidence intervals of the mean. (a) Mean reverberation times *T*_30_. (b) Mean speech clarity values *C*_50_.

Room acoustic measurement results are plotted in Figure 12. Measured reverberation times *T*_30_ are in the range of 0.12 to 0.43 s and show an increasing trend toward lower frequencies, as shown in Figure 12(a). The mean mid-frequency reverberation time according to ISO 3382-2 (2008) was calculated as *T*_30, mid_ = 0.2 s, resulting in a Schroeder frequency of approximately 281 Hz.

Calculated clarity indices *C*_50_, as defined in ISO 3382-1 (2009), with values between 7 and 29 dB, are plotted in Figure 12(b) and qualitatively show a roughly inverse curve progression compared with reverberation times *T*_30_. Expressed as intelligibility-weighted and summed single value (Marshall, 1994), these clarity indices result in a composite value of approximately 23 dB, reflecting excellent speech clarity.

**Figure 13.**
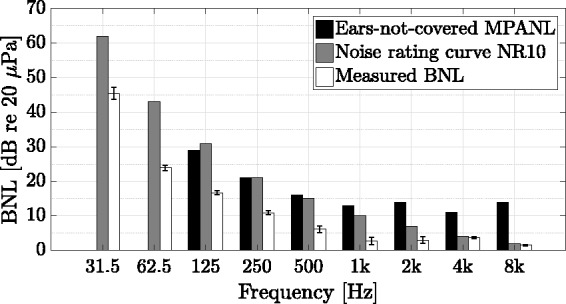
Mean BNL in the example listening environment plotted alongside ears-not-covered MPANLs, defined by ANSI/ASA S3.1 (1999), and the adequate noise rating curve NR10 (ISO 1996, 2016). Error bars indicate 95% confidence intervals of the mean. *Note.* BNL = background noise level; MPANLs = maximum permissible ambient noise levels.

Figure 13 shows measured frequency-dependent BNLs in octave bands with center frequencies between 31.5 and 8000 Hz. Values exhibit a typical increase toward low frequencies with a maximum value of 45 dB SPL in the 31.5 Hz octave band. Expressed as single values, this results in an average unweighted and A-weighted equivalent continuous sound level of *L*_Z,eq_ = 50 dB and *L*_A,eq_ = 12 dB, respectively. For comparison, normative values for the ears-not-covered maximum permissible ambient noise levels, defined by ANSI/ASA S3.1 (1999), as well as the appropriate noise rating curve NR10 (ISO 1996, 2016), are plotted additionally.

### Combined System Latency

Both measured impulse responses from the respective auralization paths were corrected for acoustic run-time of the signal emitted by the VSS, which corresponds to a subtracted delay of 256 samples (5.8 ms), given a speed of sound of 344 m/s as used in the simulation. The ASIO driver interface (RME Fireface UC, driver version 1.099, hardware revision: 133) displayed a sound-card input latency of 148 samples for a sampling rate of 44.1 kHz, equivalent to 3.4 ms, and a selected buffer size of 128 samples.

**Figure 14.**
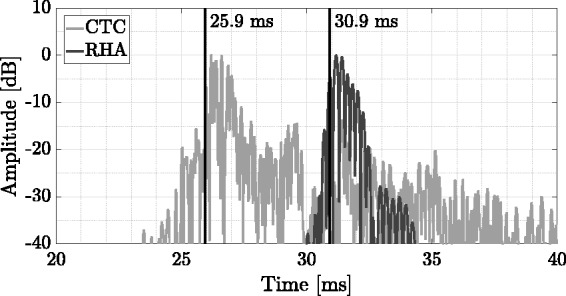
Measured static end-to-end rendering and reproduction latency. Normalized logarithmic impulse responses of both binaural paths measured from an artificial head (left-ear signal only) are shown. The impulse responses are corrected for the acoustic run time Δ*t*_propagation_ of the signal emitted by the virtual sound source, as well as the sound-card’s A/D latency. System components contributing to static end-to-end latency are shown in Figure 5, and relevant setup parameters are provided in Table 3. *Note.* CTC = crosstalk cancellation; RHA = research hearing aid.

After excluding these unwanted contributing factors, the corrected impulse responses featured static EEL values of $\Delta t_{\text{CTC}}=1,142\;\text{samples}$ (25.9 ms) and $\Delta t_{\text{RHA}}=1,363\;\text{samples}$ (30.9 ms), defining the start of the impulse response according to ISO 3382-1 (2009), cf. Figure 14. The difference between impulse response onsets confirmed the predefined relative delay of 5 ms.

In typical applications of the system, user interaction needs to be considered additionally. Therefore, the dynamic EEL, including the tracking system, must be determined. This latency was not measured but can be estimated on the basis of values provided for similar tracking systems. Teather, Pavlovych, Stuerzlinger, and MacKenzie (2009) reported tracker latencies for a different tracker hardware (Flex:C120; 120 Hz frame rate; NaturalPoint, Inc. DBA OptiTrack, Corvallis, Oregon) in the range of 73 ± 4 ms. More recently, Friston and Steed (2014) measured lower latencies derived from the preview window of the software Motive 1.0.1. Additional information provided by the author confirmed the usage of Flex 3 tracking cameras, using a frame rate of 100 Hz. Values in the latter publication rely on different latency measurement methods and resulted in a maximum value of 54.0 ms and a mean value of 50.43 ms for the tested configuration “PC 3 OptiTrack Motive Rigid Body Aero Off,” on a Windows 7 system. We take these measured values as an upper estimation of latency, although our system, presented in this article, uses tracking cameras with higher update rates, additional processing time for visual rendering of software not included. Taking into account the highest latency value reported by Friston and Steed (2014), and adding this to our measured static EEL, the dynamic EEL for the CTC path of the proposed system is expected to be below

(2) $$\begin{array}{c} {\text{EEL}}_{\text{dynamic},\text{CTC}}={\text{EEL}}_{\text{static},\text{CTC}}+\Delta t_{\text{tracking},\text{max}}=\\ 25.9\text{ms}+54.0\text{ms}=79.9\text{ms}. \end{array}$$

## Discussion

### Measurement of Spatial Transfer Functions

The magnitude spectra of both types of spatial transfer functions are unaffected by reflections from torso and head up to about 1 kHz (cf. Figure 6). Toward higher frequencies, the typical HRTF spectral magnitude pattern develops (Møller et al., 1995). For very high frequencies and ipsilateral directions of sound incidences, that is, directions between 0deg<ϕ<180deg, interferences due to pinna-related reflections lead to narrow-band notches (Raykar, Duraiswami, & Yegnanarayana, 2005; Spagnol & Avanzini, 2015), usually referred to as monaural cues. Monaural cues are relevant for the localization of elevated sound sources, especially on so-called “cones of confusion” (Hebrank & Wright, 1974; Middlebrooks & Green, 1991). These notches are naturally less pronounced in HARTF data, owing to the RHA microphones being placed behind the ear. In combination with spatial displacement regarding the artificial head’s in-ear microphones, this produces large spectral differences between the two data sets, as shown in Figure 6(c). On the contralateral side, that is, for directions between 180deg<ϕ<360deg, shadowing and diffraction effects occur, due to the head’s influence, and lead not only to a generally lower energy level and distinct spectral notches but also to spots with local energy maxima (Shaw, 1974). Figure 6(a) confirms this effect showing notches between 8 kHz and 11 kHz in the HRTF data, which are less pronounced or not present in the HARTF data set (cf. Figure 6(b) and (c)).

Differences in ILDs between HRTF and HARTF data (cf. Figure 7) are mainly rooted in the different spectral quality of the two transfer function data sets, owing to missing pinna cues in HARTF data and spatial displacement of the RHA microphones regarding the in-ear microphones, resulting in generally flatter ILD curves in HARTF data as opposed to more pronounced ILD notches in HRTF data. According to the duplex theory (Rayleigh, 1907), ILDs are utilized for localizing sound sources in the horizontal plane for frequencies above approximately 1 to 1.5 kHz (Blauert, 1997). Because of the distinct variation between the two data sets and the combined weighted usage of ILD and ITD (Macpherson & Middlebrooks, 2002), the influence on localization cannot be generalized based on given results but needs further specific investigation.

The deviation in ITDs between HRTF and HARTF data sets, as shown in Figure 8, will result in a distorted source localization when listening through RHAs. For a negative ITD deviation—as shown by the dotted gray line—the azimuth angle for a corresponding direction of sound incidence will be overestimated in playback scenarios using RHAs, compared with the perception utilizing conventional HRTFs. The converse relationship is true for positive ITD deviations between the two data sets. This simplified conclusion is merely valid on the assumption that only ITDs are used for localizing sound sources in the horizontal plane (Macpherson & Middlebrooks, 2002).

### Benchmark Analysis of the Acoustic Simulation

The benchmark of acoustic simulation showed that very high update rates for the DS part of the simulation are possible. Audibility checks for the DS, implemented as simple line-of-sight checks, are relevant in the case of an interactive situation, including moving VSSs and receivers. The filter synthesis of DS, however, is a very crucial operation for any virtual acoustic scene, as a new filter is generated for every new sample buffer for each VSS. The possibility of high update rates for both DS audibility and DS filter synthesis guarantees plausible real-time auralization of most relevant parts in acoustic simulation. For reflections, the increased computational effort leads to rather low update rates, which only allow a simulation of reflections for a reduced number of VSSs, room models with very low complexity, or simulations which may lead to potentially invalid results. If higher update rates must be achieved, the image source order can be decreased to 1, which in some cases is also an acceptable configuration for plausible auralization. For the investigated example scene, it is recommended to use Configuration A, B, or C, instead of an entire real-time simulation of reverberation updates (cf. Table 2). If Configuration A is selected, only DS audibility has to be updated, whereas reflections included in BRIR and HARRIR filters are precalculated and stored in databases.

Fast simulation updates of late reverberation are rarely required in typical auralization scenarios since reverberation does not vary substantially in conventional rooms. In addition, even in virtual reality scenarios where users are encouraged to move intuitively, translational and rotational velocities of less than 16 cm/s and 10 deg/s, respectively, were observed for most users (Lentz et al., 2007). Nevertheless, for more static scenes of simple geometry, echograms and reverberation filters should be calculated in advance and only be exchanged in case of relevant user interaction. This can be implemented applying a definition of thresholds, separately for each simulation component for translational and rotatory movement, which have to be exceeded to initiate an update (Aspöck et al., 2014). Although this does not lead to a physically correct simulation of the reverberation for all positions, it efficiently creates a plausible and interactive representation of the virtual scene. Eventually, the supervisor has to select the preferred configuration depending on experimental requirements.

In the implemented system, additional processing, like scene management and the convolution of BRIRs and HARRIRs with anechoic signals, needs to be performed by the real-time auralization engine. Using the configuration with lowest computational effort for the simulation (cf. Configuration A of Table 2), the system is able to provide a flawless auralization for three VSSs on a desktop PC (Intel Core i7-4770 @ 3.4 GHz), using ASIO drivers at a buffer size of 128 samples. For this example scenario, identical configuration parameters to the EEL measurements (cf. Table 3), were used. Both room acoustic filters, that is, BRIRs and HARRIRs, were set to a length of 44,100 samples (equivalent to 1 s at 44.1 kHz), resulting in a total of 12 convolutions for the room acoustic filters and 12 convolutions for the DS with a filter length of 256 samples. The application of longer room acoustic filters or the rendering of more than three VSSs for the given configuration, however, makes the system prone to dropouts and audible glitches, especially where there is concurrent activity, for example, by the MHA software or the experimental MATLAB interface. To improve this shortcoming and increase the possible maximum VSS count and the length of room acoustic filters, further investigations and optimizations need to be undertaken relating to the applied simulation engine.

### Combined Binaural Reproduction

#### HA-based reproduction

Distinct peaks at the ear simulator’s resonance frequencies have to be included when calculating final frequency-dependent gain values of fitting curves, to obtain the correct amount of amplification. This includes deviations with respect to sound pressure levels measured from real ear compared with measurements from an ear simulator. When using either a 2-cc coupler (IEC 60318-5, 2006) or an ear simulator (ITU-T P.57, 2009), this discrepancy is commonly referred to as real-ear-to-coupler difference, and described, for example, by Dillon (2012). For applications involving children with substantially different dimensions in ear canal diameter and length compared with the adult ear coupler or simulator, this gain mismatch can be expected to be considerably higher (Bagatto, Scollie, Seewald, Moodie, & Hoover, 2002; Fels et al., 2004).

Naturally, the choice of the ear piece during the fitting process depends on the type and degree of HL and has to be carefully arranged by the attending audiologist or HA acoustician (Dillon, 2012). However, owing to lower attenuation potential below about 2 kHz, fittings using a silicone dome ear piece would be preferable to the tulip ear piece regarding the proposed extended binaural real-time auralization system. For subjects with mild HL and good residual hearing toward low frequencies, this choice allows for perception of the external sound field, potentially facilitating the use of residual hearing capabilities.

#### External sound field reproduction

The theoretical CS in the case of matched filters, as shown in Figure 11(a), exhibits very good performance over the entire frequency range. Broadband CS values for the selected artificial head rotations are around 53.1 dB, on average, see Table 6, but are lower than those reported by Akeroyd et al. (2007) and Bai and Lee (2006). This degradation might be related to the nonideal measurement environment in which the HRTF data sets were measured for this experimental evaluation, as opposed to HRTF measurements, which are ideally conducted in an anechoic chamber. The performance drop in CS around 10 kHz is possibly linked to unfavorable spectral properties in HRTF data of the employed artificial head and their influence on the quality of resulting CTC filters in this frequency range but needs further investigation.

A considerable decrease in CS compared with the ideal CS of a CTC system, cancelling only playback HRTFs instead of playback BRIRs, had been predicted, as the four CTC is not capable of properly cancelling out distorting room reflections (Kohnen et al., 2016). Further evidence of this limitation is provided by Sæbø (2001). Although broadband CS still lies above the minimum audible CS for most stimulus types (Parodi & Rubak, 2011), it only reaches moderate to poor values below 2 kHz. As a consequence, the perception of a binaural signal is progressively reduced, because the binaural nature of the input signal cannot be satisfactorily reproduced. For frequencies above 4 kHz, however, the CS is still high enough, even in the presence of detrimental reflections.

#### Listening environment

As expected from room volume and as a result of room acoustic treatment by means of ceiling absorbers and wall panels, the measured reverberation times *T*_30_ are very low, cf. Figure 12(a). However, especially in the low frequency range, *T*_30_ increases, resulting in a low-frequency pronunciation, which affects the LS-based binaural reproduction. The detrimental effect of additional room reflections manifests itself in reduced CS, as discussed earlier and shown in Figure 11(b).

According to Hoffmeier (1996), *C*_50_ should be above −2 dB to preserve an 80% syllabic intelligibility, assuming a speaker with a directivity factor of γ = 3. In the context of speech intelligibility, where the presented material has a higher contextual predictability, this value corresponds to approximately 95% intelligibility. As the measured clarity indices in the example listening environment notably exceed this lower threshold value (see Figure 12(b)), an excellent speech intelligibility can be expected in the considered frequency range.

Measured BNLs fulfill the requirements for hearing measurements in an audiometric test room, defined by ears-not-covered maximum permissible ambient noise levels, in ANSI/ASA S3.22 (2014), and additionally lie below the NR10 curve (ISO 1996, 2016). In combination with the decoupled construction of the hearing booth (room within a room), these ambient noise conditions will allow for accurate results in audiometries and guarantee minimal distraction during listening experiments.

### Combined System Latency

Static EEL measurements resulted in values well below the minimum detectable thresholds of Brungart et al. (2005) and Yairi et al. (2006) and also below the required minimum latency for VAEs of 50 ms (Vorländer, 2007). The temporal difference between the latencies of both auralization paths, Δ*t*_RHA_ and Δ*t*_CTC_, was measured to be consistent with the predefined relative delay of 5 ms. These results show that the system’s real-time requirements are not violated by processing and reproduction of the simulated audio data, and that the relative delay of the auralization paths can be accurately controlled.

For the intended application, however, dynamic EEL, motion tracking included, has to be considered. Estimated EEL values (EEL_dynamic,CTC_ = 79.9 ms) are slightly higher than reported minimum detectable threshold values of Brungart et al. (2005) and Yairi et al. (2006) but below those reported by Lindau (2009). For application purposes of the system, these values are in an acceptable range, which is supported by the subjective impression of the system’s reactivity. Owing to higher update rates of the cameras used, and an updated version of the tracking software, the actual dynamic EEL of the system is expected to be lower.

## Conclusions and Outlook

### Conclusions

A binaural reproduction system for real-time auralization purposes, extended for applications involving HAs, has been presented. The proposed system consists of HA-based playback to reproduce simulated HA signals, which are additionally processed on a MHA platform, via RHAs and an external sound field through LSs in combination with acoustic CTC filters, in this way taking into account residual hearing capabilities of subjects with HL while also enabling auditory experiments on subjects with NH. Playback signals involved are simulated on the basis of HRTFs and HARTFs, both being measured from either an adult artificial head or individually on a dense spatial grid. The auralized scene is updated according to real-world user movements, which are captured via an optical motion tracking system. Room acoustic simulations either apply a filter set of precalculated BRIRs and HARRIRs, or calculate these filters in real-time with varying filter update rates. The entire virtual acoustic scene, including source signals, sound source levels, directivities, and trajectories, is fully controllable using a customized HAA module with MATLAB interface.

To test its validity, system properties and performance were investigated at different levels. Outcomes and recommendations are summarized in the following:

- A comparative evaluation of measured spatial transfer functions revealed considerable differences between HRTF and HARTF data sets. Differences in binaural cues in combination with decreased or missing monaural cues, as well as the different directivity pattern observed in HARTF data, will likely lead to distorted localization performance, particularly for playback of binaural signals via RHAs.
- The conducted simulation benchmark analysis showed that the DS in simulations can be updated at very high rates, while reflections and the reverberation tail have to be calculated at substantially lower update rates, which might lead to audible filter exchange artifacts. Particularly in experiments where user interaction and dynamic scenes are included, it is therefore recommended to only update those simulation parts in real time that can guarantee seamless auralization, depending on available processing resources. While this is acceptable for quite static situations, such as a classroom situation, the efficiency of applied simulation models should be increased to enable full room acoustic simulations with a large number of VSSs.
- Distinct spectral peaks and low-frequency characteristics of different ear pieces, that were observed when measuring the RHAs’ receiver transfer functions, need to be considered during the fitting process. With respect to children, it is of particular relevance to conduct individual measurements in order to factor in real-ear-to-coupler differences, owing to differing anthropometric ear canal dimensions. Based on the measured frequency-dependent passive amount of damping, we recommend the use of an open-dome ear piece for improved external sound field perception, provided that this fitting type is appropriate for the individual type of HL.
- Results of room acoustic measurements predicted very good conditions for the reproduction of VAEs over LSs in the example listening environment. Measured BNLs additionally implicate very low disturbance potential during listening experiments and facilitate accurate audiometries.
- Investigations on external sound field reproduction fidelity, using acoustic CS as performance metric, again confirmed the sensitivity of acoustic CTC systems to room reflections, even given very good room acoustic conditions. Although exhibiting very good CS values in the theoretical scenario, achieved practical CS values were only moderate to low toward lower frequencies measured in the example listening environment.
- The system’s measured static EEL lay below the required minimum latency values for an auralization of VAEs, provided in literature. Estimated dynamic EEL also exhibited values in an acceptable range and promise highly reactive auralization, potentially supporting plausible perception in case of user movement.

Based on these conclusions, the developed extended binaural real-time auralization system with an interface to RHAs represents a powerful tool and can be considered for various applications within the scope of auditory research involving subjects with HL. Because of its low hardware requirements, the system is worth considering for future use in clinical environments with limited space, enabling fitting routines with the aim of bridging the gap between HA settings adjusted under clinical laboratory conditions and the perceived, sometimes unsatisfactory real-world performance of HAs. In this context, the system provides industrial applicability for the evaluation of HA algorithms by simulating complex and problematic acoustic scenarios. Due to its transparent nature, scientific experiments can be designed and conducted efficiently, also facilitating the implementation of advanced paradigms while promoting principles of reproducible research.

### Outlook

To improve practical CS performance of the acoustic CTC system, especially toward low frequencies, further research needs to be carried out focusing on the cancellation of room reflections. For a better grounded quantification of the system’s reactiveness, dynamic end-to-end system latency will be determined through direct measurements of the motion tracking system’s latency. The sound field reproduction error in cases involving a dynamic listener is of particular interest and needs to be investigated by means of experimental evaluations. For an increased performance when auralizing complex scenes, with a high number of VSSs and computationally challenging room acoustic conditions, the applied models for room acoustic simulations will be reexamined carefully to further improve their efficiency. The performance of the proposed system will be additionally compared with other spatial audio reproduction systems which are potentially suitable for HA-related research.

In upcoming listening experiments, different spatial audio quality parameters, such as sound source localization and auditory distance perception, will be investigated. These experiments will also provide indications about the extent to which the reduced CS affects the perception of the binaural signal. How the given spatial audio quality parameters are influenced by the combined binaural reproduction approach, is of particular interest.

To show the system’s practical feasibility, speech reception thresholds using various spatial configurations of a target talker and distracting talkers will be measured under simulated room acoustic conditions. Possible investigation groups, including children and adults with HL, children diagnosed with attention deficit hyperactivity disorder, and children with a suspected central auditory processing disorder, will be tested in extensive experiments, which are already partially completed. Comparing the results to prior studies using the same paradigm, conducted under free-field conditions, will reveal the effect of plausible room acoustic simulations on speech perception and spatial release from masking.

## Notes

1 In this article, a right-handed head-related spherical coordinate system is used with its origin in the center of the interaural axis. The listener looks into negative *z* direction. Azimuth angles, given in degrees, are increasing counterclockwise with ϕ∈ℝ|0≤ϕ<360, and zenith angles, given in degrees, are defined in the range of ϑ∈ℝ|0≤ϑ≤180.

2 Although not fully released under open-source license, most parts of the RAVEN software environment are freely available for academic purposes. For further information, please contact lukas.aspoeck@akustik.rwth-aachen.de.

3 These impulse responses represent HARTFs in time domain and must not be confused with HARRIRs.
